# Use of a quantitative data report in a hypothetical decision scenario for health policymaking: a computer-assisted laboratory study

**DOI:** 10.1186/s12911-021-01401-4

**Published:** 2021-01-28

**Authors:** Pamela Wronski, Michel Wensing, Sucheta Ghosh, Lukas Gärttner, Wolfgang Müller, Jan Koetsenruijter

**Affiliations:** 1 Department of General Practice and Health Services Research, Heidelberg University Hospital, Im Neuenheimer Feld 130.3, 69120 Heidelberg, Germany; 2 Scientific Databases and Visualization Group (SDBV), Heidelberg Institute for Theoretical Studies – HITS gGmbH, Schloss-Wolfsbrunnenweg 35, 69118 Heidelberg, Germany

**Keywords:** Health policy, Decision-making, Data use, Reading behavior, Evidence-based health policy, Eye-tracking, Laboratory

## Abstract

**Background:**

Quantitative data reports are widely produced to inform health policy decisions. Policymakers are expected to critically assess provided information in order to incorporate the best available evidence into the decision-making process. Many other factors are known to influence this process, but little is known about how quantitative data reports are actually read. We explored the reading behavior of (future) health policy decision-makers, using innovative methods.

**Methods:**

We conducted a computer-assisted laboratory study, involving starting and advanced students in medicine and health sciences, and professionals as participants. They read a quantitative data report to inform a decision on the use of resources for long-term care in dementia in a hypothetical decision scenario. Data were collected through eye-tracking, questionnaires, and a brief interview. Eye-tracking data were used to generate ‘heatmaps’ and five measures of reading behavior. The questionnaires provided participants’ perceptions of understandability and helpfulness as well as individual characteristics. Interviews documented reasons for attention to specific report sections. The quantitative analysis was largely descriptive, complemented by Pearson correlations. Interviews were analyzed by qualitative content analysis.

**Results:**

In total, 46 individuals participated [students (85%), professionals (15%)]. Eye-tracking observations showed that the participants spent equal time and attention for most parts of the presented report, but were less focused when reading the methods section. The qualitative content analysis identified 29 reasons for attention to a report section related to four topics. Eye-tracking measures were largely unrelated to participants’ perceptions of understandability and helpfulness of the report.

**Conclusions:**

Eye-tracking data added information on reading behaviors that were not captured by questionnaires or interviews with health decision-makers.

## Background

Decisions in health policy are influenced by a mix of factors, such as the prevalence and urgency of health needs, values and preferences in the population, the options for treatment and care, and the available healthcare capacity [1]. Many of these issues can be measured and quantified for specific scenarios, for instance, healthcare planning in a geographical region or a defined population. Quantitative data reports aim to help decision-makers to make choices that are most consistent with their values and interests. Empirical evidence from selected Australian public health decision-making bodies suggests that internal data and reports are the most frequently used, while research evidence is used the least [2]. Research from the US American health policy context points in a similar direction [3]. While quantitative data reports are widely produced, little is known about how they are actually read and used by health decision-makers [4]. Previous work on evidence-based health policymaking (HPM) focused on barriers and facilitators of evidence use [4–7] and aimed to “bridge the gap” between the production of evidence by scientists and its use by decision-makers [8–10]. This research focus is also discussed critically because it bears the risk of neglecting other legitimate factors of policymaking, such as negotiating conflicting values within societies [11–13]. A systematic review identified ‘*availability and access to research/improved dissemination*’ as the most crucial barrier and facilitator at the same time [5]. As general format policymakers e.g. seem to prefer evidence summaries over plain systematic reviews [14]. Further, there are suggestions to use report formats, which take account of different needs that vary among policymakers and time. For example, the 1:3:25 format was discussed. It combines brief summaries e.g. for those who preferably read an abstract and conclusions and more detailed information, including more methodological background [15]. How individuals actually read reports after they finally became available and accessible may indicate what information they prioritize for their decision under limited time.

Previous applications of eye-tracking to explore the reading of quantitative data reports in the context of HPM are rare. Vass et al. explored potential uses of eye-tracking to better understand the results delivered by discrete choice experiments in the context of a breast screening program. They used eye-tracking data to validate self-reported attribute non-attendance and to explore the impact of risk communication on respondents’ decision-making strategies [16]. King et al. used eye-tracking to explore clinicians’ reading behavior of electronic health records from patients receiving critical care. Eye-tracking data proved to be a potential alternative to manual selection for the purpose of training a model that learns an electronic health records system in displaying relevant information [17].

From a rational choice perspective health decision-makers may be expected to critically assess the quality of the provided information and to incorporate the best available information into their decision-making process. However, decision-makers frequently do not apply a fully rational approach but optimize decision-making within constraints (‘bounded rationality’) [18, 19]. The present study uses innovative methods to explore how (future) decision-makers in healthcare actually read and use data reports for making decisions.

The planning of nursing care for people with dementia in a defined geographical area is a good example of the complex process of HPM, which will be used in the presented research. Given the expected rise of the numbers of these patients in Germany from 1.7 million in 2016 [20] to a range between 1.9 and 2.4 million by 2030 [21], more nursing care capacity seems needed. However, the predicted numbers of patients in the future are associated with uncertainty. Increasing nursing home capacity is one obvious option for more nursing care, but this is relatively expensive and may not meet patients’ preferences. Ambulatory nursing is an alternative option, which is less costly and enables that patients remain at home. A final option is supporting (also financially) relatives who nurse these patients at home. This option requires the least resources for nursing care, but could imply a high burden for relatives.

Many decision-makers in healthcare have a background in one of the health professions, which usually provided them little explicit training to interpret quantitative reports for HPM, although training does not provide a guarantee for the ability to understand and interpret. For instance, an international sample of 531 clinicians and trainees from the fields of family medicine and internal medicine was found not understanding well information on treatment effects from meta-analyses [22]. Bramwell et al. found in their experiment that less than the half of participating midwives and obstetricians interpreted probabilistic screening information correctly [23]. Lopez et al. reported similar results examining numeracy and graph literacy among nurses [24]. Presentation methods, such as graphs and tables, may influence the interpretation of quantitative data. For instance, specific types of graphs may be best understood [25–28]. The other way round, the presentation format of research findings may lead to misinterpretations [29, 30].

This paper aims to describe and assess the reading behavior of quantitative data reports by (future) health policy decision-makers, who were requested to decide on the planning of nursing care for people with dementia.

## Methods

### Study design

We conducted a computer-assisted laboratory study comprising a computer-based quantitative data report and observational measures (eye tracker, questionnaire, semi-structured interview). Due to the exploratory character of the study, the design was observational. This study was approved by the research ethics committee of Heidelberg University Hospital (number of ethics approval: S-857/2018).

### Study population

The study population included current and potential future healthcare professionals who are involved in local HPM, either as the main task or alongside other tasks. These included employees of health insurance schemes, physicians in an executive position, employees of health facilities working in administrative departments, and scientists from the field of health services research. The sample was limited to persons who were at least 18 years old and had good knowledge of the German language. Furthermore, the eye-tracking measures required that participants were not blind nor had implanted artificial lenses. Exploratory studies lack prior knowledge to calculate the realistic sample size. Therefore, no specific sample size was calculated [31–33]. Instead, we orientated the sample size on the feasibility and a balanced size of the participating groups. This resulted in a total sample size of 40 to 60 participants. For our sample, we recruited participants within three different groups: starting students, advanced students, and professionals.

Starting students were recruited from the first two semesters of the study program human medicine and the bachelor program interprofessional healthcare (IPG) from the medical faculty of the University of Heidelberg. The IPG students combine a university program with vocational training in nursing or an allied health profession such as physiotherapy or speech therapy. Advanced students were recruited from the Master of Science program in health services research and implementation science in healthcare, and the seventh semester and upwards of medical students. Students were invited to the study by an email, which was sent by the study program coordinators or secretary. Additionally, posters on campus and short presentations in bachelor’s and master’s classes were used to approach students. At the time of recruiting 364 first-year students and 1520 advanced students were enrolled in eligible study programs and semesters [34].

Professionals were selected purposively from the working environment of the study team and the region of the research setting. This included former project partners involved in public health administration, colleagues from inside and outside of the study team’s organization, and persons involved in HPM in the study site’s region not personally known to the study team. The latter group was identified via the web page of the communal health conference (CHC) of the study team’s administrative district. The web page provided information on involved organizations and their representatives in the communal working group for long-term care. The CHC is a networking platform for local health system stakeholders. They can organize themselves in communal working groups on an agreed topic to further develop local healthcare in this area. Our study’s intervention is embedded in a hypothetical scenario that relates to the working group for long-term care. Professionals who were found via an internet search were sent study invitations by post (n = 8) and others were invited via email (n = 20).

An equal representation of the three groups was pursued. The study took place in a laboratory setting, which required traveling to the university’s campus. Students were paid a remuneration of €15 after participation and professionals were offered compensation for their travel costs.

### Data collection and research setting

Data was collected in the Eye Tracker Laboratory, Scientific DataBase and Visualisation group at the Heidelberg Institute for Theoretical Studies (HITS) between April 2nd and November 20th, 2019. Before data collection, participants were informed in written and oral form about the study context, the procedure of the data collection, and data security. Participation was voluntary and study withdrawal was possible at any time of the study until collected data was anonymized.

Instructions were given to participants verbally before data collection and in a written form on the computer screen. In each participant, four different measurements were conducted: computer-assisted eye-tracking during the performance of the task, two computer-assisted questionnaires, and a face-to-face interview at the end. All measurements were intended to last between 60 to 90 min for each participant. During data collection, two members of the study team were present in the laboratory. One study team member was giving instructions prior to data collection and conducted the interviews. The other study team member, experienced in the scientific use of eye trackers, performed a 5-point calibration before each data collection to achieve a satisfactory accuracy of data acquisition. If calibration was not possible, the experiment was not conducted.

For the eye-tracking, we used *Tobii-X1 light* [35], a desktop-mounted and binocular eye-tracker. Eye-tracking data was collected with Tobii eye-tracker software (version 3.4.8). The device directs infrared lights towards the center of the eyes which results in pupil and corneal reflection patterns. These reflection patterns are detected by image sensors and used to compute the eyes’ position and gaze points. This version of Tobii eye tracker produces around 30(± 2) frames per second (FPS). Data from both eyes were used calculating average values for eye-tracking measures. The recommended distance between a participant’s eyes and eye tracker device is circa 65 cm and was captured once for each participant during calibration before data collection [36]. Further, participants were instructed to keep this distance. The laboratory was a well-lit room with windows covered with curtains. In case participants were wearing glasses, curtains were opened, to let natural sunlight into the room. Interviews were audio-recorded and transcribed.

#### Intervention

The function of the intervention in this study was the simulation of a decision scenario in the field of HPM, in which a quantitative data report is given to participants and the task to make a decision.

Before the report was displayed, the hypothetical decision scenario was presented to participants on the computer screen. In brief, participants were part of a communal working group on regional long-term care. There, participants were asked to remain in their current roles of their real lives e.g., student, nurse, physician, or a child of a parent in need of care, as participation in this working group, in reality, is open for all local citizens. The hypothesized task of this working group was to advise the local district administrator on the use of additional funds for long-term care, especially for persons with dementia. It was described that the working group agreed on a preselection of options for the use of additional funds earlier. For the next meeting of the working group it was planned that members agree on one option for the use of funds to be recommended to the local district administrator. As a member of the working group, study participants were asked to prepare themselves for this meeting, where they would advocate one of the preselected options. For study participants this implied that they needed to make their choice individually. Further, this implied that the decision comprised only one step, i.e., there was only one decision to make, which only had to be made once. As support for their meeting preparation, participants were given a quantitative data report about the supply and demand for long-term care in the community prepared by the working group. A specific aim for the decision was not given in the scenario description. It was described that the interest of the working group was to use additional funds where they are needed most. A summary of the decision scenario is given in Table 1.

**Table 1:** Summary of decision scenario

Decision component	Specification
Decision problem	One-step: How to spend additional funds for long-term care in community?
Given options	A. more support for informal carers B. more ambulant nursing capacity C. more nursing home capacity
Potential consequences	A: lowest cost, most people reached B: medium cost, medium number of people reached C: highest cost, least people reached
Decision maker	Individual (study participant makes decision alone)
Aim/goal	Not defined explicitly (implicitly, aim of working group stated in scenario description: ‘use additional funds where they are needed most’)

The quantitative data report (Additional file 1) was displayed on the computer screen together with a tick box, which required participants to choose one of the proposed options. Additionally, there was the option to make comments. Participants were instructed to spend no more than 20 min on the decision task and the reading of the report. The presented report was written in German, thirteen pages long (4111 words) and was structured like a short project report comprising a title page, a table of contents, an introduction (circa 1.5 pages), a methods section (circa 3.5 pages), a results section (circa 4.5 pages), and a discussion and conclusion section (circa one page). To access all pages of the report, participants had to scroll down. The introduction included a short description of the three options which were preselected by the working group for the use of additional funds for long-term care in the community (see Table 1). Quantitative data presented in the report were real descriptive figures on current and projected demand and supply of long-term care services in the region of interest based on secondary data analyses of real data [37].

#### Measures

We used three different methods of data collection: eye-tracking based, questionnaires, and interviews.

##### Eye-tracking

Based on the eye-tracking data, we extracted five measures and calculated mean values over report sections and the three report figures. Time spent (in minutes) to read the report and complete the task was documented on the basis of recordings. Three eye pupil-based measures were extracted: diameter (in mm), dilation (in mm), and response (in mm). The diameter is generally used as a measure of pupil size. Dilation describes the increase of pupil size and was measured as the difference between largest and smallest pupil diameter within an individual during processing of a report section. Pupillary response was used to summarize changes in pupil size resulting from dilations and constrictions (decrease of pupil size) by summing these two ways in pupil size change. These three pupillometric measures are considered to be indicators of cognitive load while performing the task [38, 39]. The fifth and final eye-tracking measure was average fixation duration (in milliseconds), which is used as an indicator of attention for processing information from the report [40].

For descriptive purposes, we also determined ‘heatmaps’ of reading behavior with respect to attention, which visualize the fixations during reading the report [41]. More fixations to a report part correspond to more attention to it and were indicated by red colors, fewer fixations were indicated by green colors [42]. As the range of fixations represented by colors was defined individually for each participant, a visual appraisal of different color shades is only possible within one heatmap and not comparable interpersonally.

To assess that fixations in our data were the result of attention rather than daydreaming, we visually estimated white space fixations by an analysis of gaze plots from all participants. Gaze plots show the location of gaze points and how much time spent on looking, that is, the duration of fixations by circles plotted over the stimulus in the time sequence fixations occurred.

##### Questionnaire

For this study, we developed a questionnaire. An English version of the questionnaire is available in Additional file 2. Participants were asked to document individual characteristics that we hypothesized to influence the reading and decision-making task: demographic information, educational background, and practical experience in healthcare. We measured participants’ tolerance of ambiguity as data in reports comes with a certain amount of uncertainty. This was done by the validated 8-item test [43], using adapted wordings [44]. Participants had to rate each item on a 6-point scale from ‘*absolutely true*’ to ‘*absolutely not true*’ where a higher score indicates higher tolerance of ambiguity. Further, we tested participants’ adequate understanding of the information presented by graphs in the quantitative data report using 5 items. The question type of the items was adopted from Galesic et al. who tested general graph literacy [45]. For our study, scoring was conducted by summing the number of correct answers, which results in a range of 0 to 5 possible points. Statistical numeracy or risk literacy was measured using the validated Berlin numeracy test of the 4-item paper and pencil version in German language [46]. A score was calculated by taking the proportion of correct answers, resulting in a range of 0 to 1. A proportion of 0.55 can be regarded as an average for “highly numerate individuals” [46]. To support assessing the quality of eye-tracking data, we asked participants about their use of visual aids such as glasses during the report reading task. Finally, participants were asked to assess each section of the report (introduction, methods, results, discussion and conclusion) according to their understandability and helpfulness during the decision-making task, both on a 10-point Likert scale from 1 ‘*not helpful at all*’ to 10 ‘*very helpful*’.

##### Interviews

To explore the experiences of participants concerning completing the task, PW and LG (both economists by training) conducted face-to-face interviews with the use of a semi-structured question guide. Interview questions were developed by the authors. The question guide was revised after six participants to encourage study participants to speak more overtly about their experiences in general and more about the way they had read the report. For the revision of the question guide, the authors consulted two colleagues, both of them are researchers (sociologist and health scientist) experienced amongst other things in developing question guides for qualitative research. An opening question was added to the question guide and all questions were reformulated. The final version of the guide was translated from German to the English language and can be found in Additional file 3. Interview transcripts were not returned to participants for correction or comments.

### Analysis

Participants were the unit of all analyses. Questionnaire and eye-tracking data (after data preparation in Tobii eye-tracker software) were analyzed using IBM SPSS Statistics Version 25. Descriptive analyses were performed to report on the appreciation of report sections given by questionnaire data as well as fixation based and pupillometric data given by eye-tracking. Additionally, Pearson correlations were calculated to explore the relation between fixation, pupillometric, and questionnaire measures for report sections separately. Considering the explorative nature of the study, we regarded a *p* value < 0.10 as significant.

For analyzing the interviews, a qualitative content analysis was conducted to explore reasons mentioned by participants on why they gave more or less attention to a report section for decision-making and to identify other aspects of the decision-making than the information provided in the data report. We used a conventional approach, where categories (in our case: mentioned reasons) are rather derived from collected data than theory [47]. The qualitative content analysis was conducted by 3 of the authors, whereby 2 of these authors were involved in data collection. First, relevant text passages were extracted from interview transcripts of all participants using ATLAS.ti version 7.5.10. Simultaneously, extracts were coded with the concerned report section. To increase interrater reliability, a coding plan was developed. Following the approach for qualitative content analysis of Gläser and Laudel [48], all further steps were conducted apart from transcripts and only on coded extracts. The next steps were a) paraphrasing, b) formulating short forms of paraphrases, and c) categorizing short forms. Paraphrasing meant to reduce extracts to the core of the statement [49]. Formulating short forms of paraphrases meant reducing extracts to a statement not relating to the report’s content. In case short forms appeared synonymous in content, they were aggregated. Finally, the coding team grouped short forms into categories referring to the short forms’ content. For each category of the analysis of reasons for attention to the report’s sections quotations from interviews were selected to exemplify the meaning of a category.

A pilot study of the whole data collection procedure was conducted with employees from the Heidelberg University Hospital (n = 3) to test and, if necessary, revise the measures and to ensure technical functionality of the eye-tracking software in combination with the survey tool.

## Results

In total, 46 persons (students 85%, professionals 15%) participated in the study (see Table 2). The targeted number of participating students was reached, though not as balanced among starting (circa 20%) and advanced (65%) as planned. Due to the burden of the intervention and data collection, it appeared hard to recruit professionals, resulting in a response rate of 22%. Moreover, the ones willing to participate were already working at the same campus and known by the authors. All participants provided data through the questionnaire, eye-tracking, and the interview. The results show that all participants had some practical experience as a healthcare provider. Risk numeracy was moderate with on average 59% correct answers. The decision task to choose an option for the future use of additional funds for long-term care resulted in a choice for ‘more nursing home capacity’ by more than two-thirds of participants (67.4%), while increasing ambulant nursing capacity was chosen by 9 participants (19.6%) and more support for informal carers by 6 participants (13.0%).

**Table 2:** Study population/individual characteristics

N = 46	n	Mean/%	SD*
Sex (% female)	37	80.4	
Age (mean)		25.74	5.42
Field (%)			
Medicine	27	58.7	
Health sciences (graduate & undergraduate)	16	34.8	
Other	3	6.5	
Level of expertise (%)			
Starting	9	19.6	
Advanced	30	65.2	
Professional	7	15.2	
Tolerance of ambiguity (range between 1 and 6)		3.53	0.68
Risk numeracy (range between 0 and 1)		0.59	0.36
Decision (How to spend additional funds for long-term care in community?)			
Option A: support for informal carers	6	13.0	
Option B: ambulant nursing capacity	9	19.6	
Option C: nursing home capacity	31	67.4	

*SD, standard deviation

The visual analysis of gaze plots showed only a few white space fixations: 1.2% of total fixations were white space fixations. They mostly (1.0% of total fixations) occurred on the blank right side of the report picture during scrolling.

### Heatmaps

**Fig. 1:**
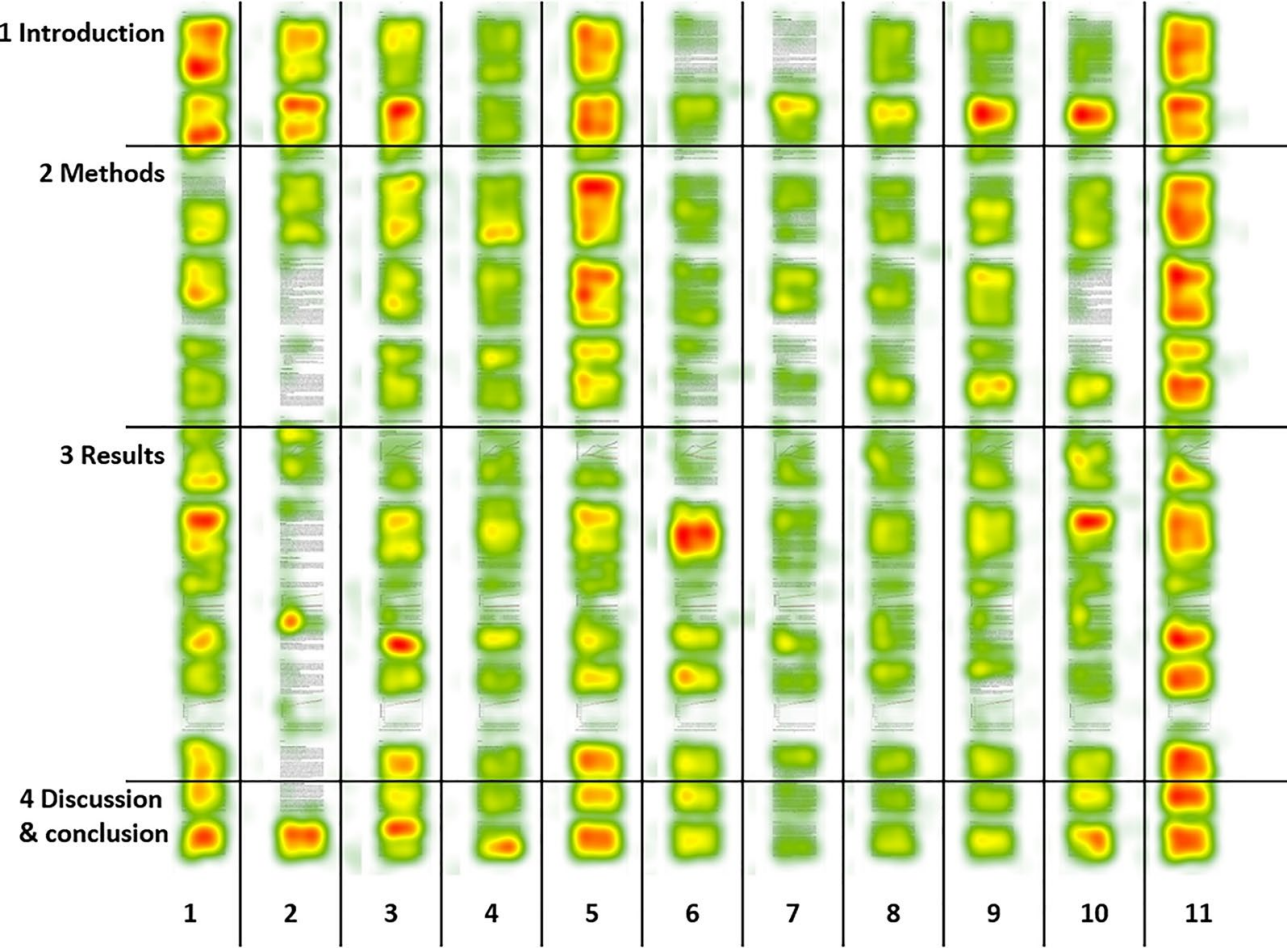
Heatmaps of 11 participants (columns) after reading the data report (rows), scaled by fixation duration. Average fixation duration in seconds over the 11 participants whose heatmaps are displayed above: red: 0.31-more/yellow: 0.30–0.24/green 0.23-less

Figure 1 shows illustratively the heatmaps of 11 randomly chosen participants. Heatmaps of all 46 participants are presented in Additional file 4. The heatmaps illustrate which report sections were given more visual attention than others by a participant. At a first glance at all 46 heatmaps, they show variation in attention both between report sections and between participants. There is a group of heatmaps, which shows more fixations in favor of some report parts like, for example, heatmap 2 (Fig. 1). In another group of heatmaps (e.g., heatmap 11 in Fig. 1) all report sections seem to be appreciated similarly. Almost all heatmaps indicate a predominant focus on the second part of the introduction, while the third figure of the report almost at the end of the results section was hardly looked at.

#### Quantitative appraisal of report sections

Table 3 descriptively apposes measures from eye-tracking and the questionnaire (perceived understandability and helpfulness for decision) arranged by report sections and the three figures displayed in the results section. Additionally, a graphical presentation of the two eye-tracking measures time spent and average fixation duration is provided in Additional file 5.

**Table 3:** Feedback on report sections, all measures in mean values with [standard deviation], n = 46

Report section and length* (in %)	Eye tracking					Questionnaire	
	Time spent (in minutes)	Average fixation duration (in ms)	Pupil diameter (in mm)	Pupil dilation (in mm)	Pupillary response (in mm)	Understandable (1 to 10)	Helpful for decision (1 to 10)
Introduction 18.8	2.9 [1.4]	433 [527]	2.71 [0.29]	0.016 [0.007]	0.033 [0.013]	9.0 [1.0]	6.2 [2.5]
Methods 32.4	3.7 [2.5]	380 [452]	2.70 [0.28]	0.017 [0.007]	0.034 [0.015]	7.0 [1.9]	4.6 [2.1]
Results 37.2	4.9 [2.3]	430 [338]	2.70 [0.29]	0.017 [0.007]	0.034 [0.014]	8.1 [1.5]	7.6 [1.6]
Figure 1 2.0	0.4 [0.2]	600 [875]	2.72 [0.27]	0.017 [0.007]	0.034 [0.014]	8.2 [1.8]	6.7 [2.5]
Figure 2 2.0	0.3 [0.1]	447 [434]	2.70 [0.32]	0.018 [0.009]	0.036 [0.019]	8.0 [2.0]	7.4 [2.3]
Figure 3 1.2	0.1 [0.0]	402 [559]	2.67 [0.24]	0.020 [0.009]	0.038 [0.017]	8.3 [1.6]	6.4 [2.8]
Discussion 11.6	1.9 [0.8]	589 [941]	2.69 [0.31]	0.018 [0.008]	0.035 [0.015]	7.8 [1.2]	6.4 [2.3]

**Fig. 2:**
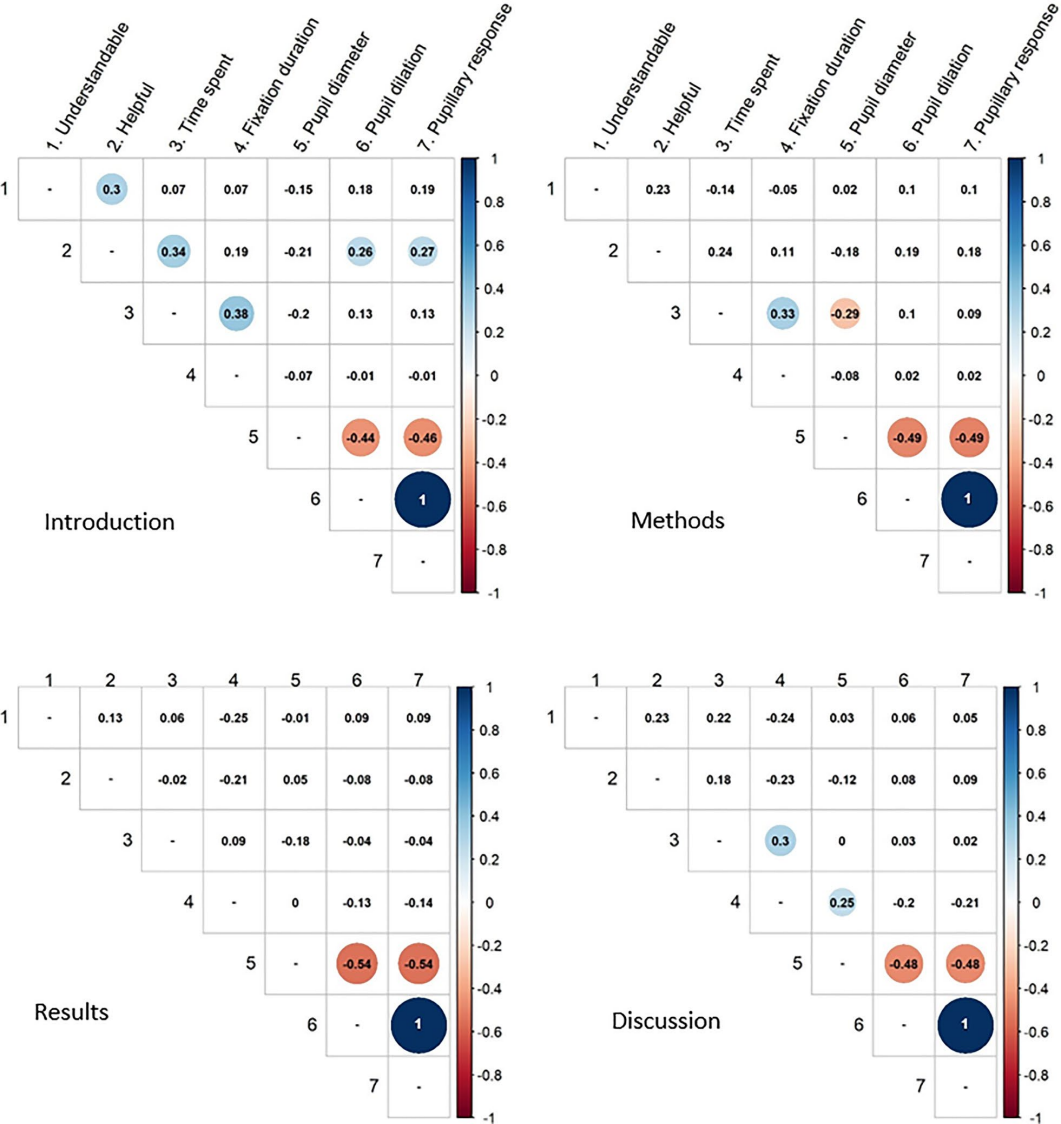
Pearson correlations between questionnaire and eye tracking based measures appreciating report sections. Questionnaire based measures: understandable, helpful for decision. Eye tracking measures: time spent, pupil diameter, pupil dilation, pupillary response. Colored figures: *p* < 0.10

*Length of report sections is approximated by percentage of words from sum of words over all report sections (n = 4042). For figures 1 to 3 words in the labelling of the axes and in captions were counted, each graph was counted as one word

On average, participants spent 13.9 min with a standard deviation of 4.9 min for reading the report. The pre-set maximum reading time limit of 20 min was reached by 4 participants. The time spent on reading a report section corresponded roughly to the length of the respective sections. This indicates that, on average the time spent per report section was in the same order of magnitude. The standard deviation for time spent was highest in the methods section, suggesting that how much time participants spent for a section varied most in this section. Time spent on the figures varied between 0.1 (*report* figure 3 *with low data density*) and 0.4 min (*report* figure 1 *with high data density*). Average fixation duration showed the same pattern with a value of 600 ms for figure 1 and 402 ms for figure 3. Among report sections, the average fixation duration was only 380 ms in the methods section, whereas for the discussion and conclusion section it was about 600 ms. Pupillometric measures did not show much variation among report sections. Participants perceived all report sections as understandable with values ranging from 7.0 for the methods part to 9.0 for the introduction. The methods part was perceived as rather not so helpful for decision-making (4.6), while the results section was perceived as relatively helpful (7.6).

Looking at the correlations between measures (Fig. 2), 3 out of 40 pairings of measures from different data sources (questionnaire and eye-tracking) were correlated statistically relevant (*p* < 0.1). There were moderate positive correlations in the introduction section between reported helpfulness: Participants felt that the introduction was more helpful if they had more time spent on reading it (r = 0.34) and if pupil dilation (r = 0.26) and pupil response (r = 0.27) showed higher values. Further correlations referred to measures of the same data source as described: The introduction section was perceived as helpful if it felt understandable to participants (r = 0.30). Across all four report sections, the strongest correlations (r = 0.44 and higher) were found between the three pupillometric measures. Across the three report sections introduction, methods, discussion and conclusion, statistically significant correlations were found between the eye-tracking measures average fixation duration and time spent, on a similar moderate level (r = 0.38, r = 0.33, r = 0.3). Two further correlations were only found for single report sections between pupil diameter and other non-pupillometric eye-tracking measures: In the methods section the time spent and pupil diameter correlated negatively (r = -0.29), whereas in the discussion and conclusion section pupil diameter correlated positively with average fixation duration (r = 0.25).

### Qualitative analysis

The mean interview duration was 17 min. The shortest interview took 7 min, the longest 31 min.

The qualitative content analysis identified 29 distinct reasons participants mentioned to explain why they gave more or less attention to a report section during the reading and decision task. Identified reasons were grouped into four categories: type of information use, perceived understandability, decision, and expectations towards report sections. Table 4 summarizes the identified categories, reasons or themes, and the report sections these themes are related to. All categories relate to all four report sections, some themes relate to only one report section, while others refer to more than one or all report sections. Furthermore, Table 4 shows the direction of attention to a report section, i.e., whether a theme was related to more or less attention to a report section.

**Table 4:** Reasons for report section attention identified by qualitative content analysis

Category	Theme (report section theme relates to)*, **	Description of category	Illustrative quote per category
Type of information use	+ establish comprehension (3, 4) + facilitates reflective thinking (1, 3, 4) + form an opinion based on given information (3, 4) + learn about authors' view (4) + personal relation to topic (3) ± previously made choice was confirmed (2, 3, 4) ± credibility of text part is important (2) get overview (1, 3, 4)	Themes describe participants’ purposes of information use and mostly relate to importance of a report section for decision task Category addressed by most participants	*“[…] I looked through the conclusion to see whether I did not miss anything, any important information I did not read, yet.”* (participant 20, advanced student)
Perceived understandability	+ text part was short (4) + written understandably (4) + figures enhance comprehension of contents (3) ± figures not understandable (3) ± figures quickly understandable (3) ± no link between information and decision (1, 2, 3) − more information provided then needed (2) − too little previous knowledge for understanding the information (2)	Themes reflect understandability of report sections in connection with more or less attention to a report section Category addressed by some participants	*“A graphic provides a nice overview and oneself can, you can see what the development is and so on, you do not need to look at the corresponding text, this saves time when one is under time pressure […].”* (participant 10, advanced student)
Decision	+ figures generally important (3) + information helpful for weighing up options (3) + text part contains important information (3) + text part relevant (1, 2, 3) ± information less important under time pressure (1–4) ± text more important than figures (3) ± text part not relevant (1, 2) − already decided before reading (3, 4)	Themes relate to decision-making process Category addressed by most participants	*“I knew, I only have 20 min, and whether this is routine data or data from a primary data collection is not so important for me, for such a spontaneous decision, this may not be a perfect answer, but in that situation it was not so important for me and therefore I did not read it so much.”* (participant 27, advanced student)
Expectations towards report sections	+ desired information was given (1, 4) ± other information desired (2–4) − expected information (3, 4) − information already known from previous knowledge (1, 2, 3) − information already known from report (3, 4)	Themes relate to participants’ expectations and anticipations towards information given in report sections Category addressed by some participants	*“Well, actually I was looking for information on effectivity of measures and I did not find it. Therefore, I thought ‘okay I will glance at the graphics, wanted to scroll down to the information I actually was looking for, which so to say, never came.”* (participant 42, professional)

*Report sections are indicated by their numbering: 1. Introduction, 2. Methods, 3. Results, 4. Discussion & conclusion

**Reason or theme was reported to either result in more (+) or less (−) attention to report section or some participants reported a reason or theme to result in more and others to less attention (±)

#### Type of information use

Most participants described what they did or intended to do with the information they gathered from a certain report section, i.e., how they used the information. These reasons were mostly related to the importance of a report section for the decision task, such as using it to form an opinion or getting an overview. Some participants mentioned that they used the methods section to assess the credibility of other report parts. However, this reason was mentioned by both, participants who told that they gave more attention to the methods section, as well as participants who gave less attention to it.

#### Perceived understandability

When participants stated their reasons for giving more attention to a certain report section than to others, some also reflected the understandability of report sections. This argument was used both to explain why more and why less attention to a report section was given. Participants who reported that the figures in the results section enhanced their comprehension of the section’s content mentioned that they gave more attention to these figures. Participants reported that they gave less attention to figures, when they perceived them as not understandable or when they could not link them to the decision task. Considering the limited time for reading, some participants appreciated that the figures in the results section provided a lot of information in a condensed and clearly represented way to be captured fast. Therefore, less time was spent on reading the text of the results section, and more time was given to the figures.

#### Decision

Reasons for giving attention to a certain report section were by most participants directly linked to the decision-making process. Throughout participants, each reason could be related to either giving more or less attention to a section. Participants who mentioned that figures were generally important to them, who perceived provided information as helpful for weighing up options, or felt that a report section contained important information reported to give more attention to specifically the results section. Being perceived as relevant for decision-making was another reason for more attention and applied to all report sections except the methods section. In turn, some participants perceived the methods section as not relevant for decision making and therefore gave less attention to it.

#### Expectations towards report sections

The last category we identified among the mentioned reasons for giving attention to a report section was the expectation towards a report section. Participants stated that they anticipated the given information of a text part or felt that the information they scanned through was already known to them, either by previous knowledge they had before reading the report or from the information they gathered in an earlier processed part of the report. Participants who reported these self-observations also stated that they gave less attention to report parts from which they did not expect to gather new information. These expectations were mostly related to the results and discussion and conclusion section.

Besides the information given in the report, there were other aspects, which participants reported to have influenced their decision-making. The qualitative content analysis resulted in three types of categories and twelve subcategories presented in Table 5. *Relationship/domestic environment* was the most frequent subcategory, as it was mentioned by 31 participants. Here, participants reflected on the benefits and burdens homecare may mean for the relationship between persons in need of care and their caring dependents as well as the consequences of a domestic environment in general. Participants observed in their own families and other people’s families that caring dependents were overburdened with providing informal care. On the other hand, participants observed that persons in need of care felt supported and strengthened by their dependents. The second most common subcategory was *preference or attitude* which participants described to have considered while making their decision. Also, many participants mentioned that their *experience in private environment* was another important aspect of making their choice. The most common perspective which participants included in their decision-making was at a societal level, such as thoughts about opportunity costs for the society which may result from informal care (option A).

**Table 5:** Aspects included in decision-making other than report identified by qualitative content analysis

**Source** (Categories under this heading refer to other sources besides the data report which participants mentioned to have included in their decision-making)	**Perspective taken** (These categories describe the perspective, from which participants reported aspects included in their decision-making)	**Content of aspect** (These categories summarize the content of other aspects which participants reported to have included in their decision-making)
**Preference or attitude** (e.g., general preference of home care)	**Society** (e.g., thoughts about opportunity costs for society which may result from informal care)	**Professionalism** (e.g., quality of professional care is higher than informal care)
**Education** (e.g., knowledge aquired at university)	**Nursing staff** (e.g.,thoughts about working conditions of nursing staff)	**Capacity** (e.g., nursing homes are full)
**Experience** * in job environment* (e.g., in nursing home) * in private environment* (e.g., from dependents)	**Concerned persons** (in need of care and dependents) (e.g., will of persons in need of care should be considered)	**Relationship/domestic environment** *benefits* (e.g., dependents give support and strength to person in need of care) *burdens* (e.g., dependents are overburdened with informal care)
**Other** (e.g., information obtained from the media)	**Oneself** (e.g., how one wants to act oneself in the future)	**Reservations about nursing home** (e.g., persons in need of care receive insufficient care)

## Discussion

This study described the use of quantitative data reports by (future) health policy decision-makers by observing them with innovative methods while reading and deciding. Our laboratory experiment shows that (future) health decision-makers spend equal time and high-level attention on all parts of a presented quantitative data report, but were on average less focused when reading the methods section. The methods section also showed the most variation between the participants in how much time was spent reading. The participants’ perceptions of understandability and helpfulness of the sections in the report were hardly correlated with measures based on eye-tracking. This may indicate that the latter could add additional information that helps to understand the reading of quantitative data reports by health decision-makers. The qualitative content analysis showed that reasons for attention to a report section can strongly vary and that persons while reading and deciding have also other aspects in mind, which go beyond the information provided in a report. This illustrates that cautiousness in interpreting eye-tracking data is important.

A strength of this study is the combination of using interviews, questionnaires, and eye-tracking providing new insights and understanding on how quantitative reports are read which would not have been possible in separate studies. Some limitations are related to the study sample. Recruiting professionals to the laboratory setting proved to be difficult. Therefore, most of our professionals were selected pragmatically and as scientific researchers only indirectly involved in HPM. Scientific skills as knowledge of methods might be lower in the normal population of decision-makers at non-academic institutions. Risk numeracy in our study population can be regarded as average for this high literate group and was found to be on a similar level as in a sample of third-year medical students and general practitioners [50]. Moreover, although sufficient for our explorative approach, the sample size did not allow us to find small effects, resulting in a few statistically significant results. Also, we have to take into account that the laboratory setting including the hypothetical decision scenario might not completely reflect normal conditions. There were no real consequences to the chosen decision and we noted that some participants might have put more effort into reading the whole report because of social desirability. On the other hand, the laboratory provided a stable environment for data collection controlling for environmental factors influencing decision-making and eye-tracking data.

In our study, the eye-tracking-based measures seemed to provide additional information, which was not captured in the questionnaires and interviews.

The study has several implications for health policy and future research. First, it describes the reading process in the context of decision-making. Heatmaps together with a low percentage of white space fixations, fixation duration, time spent per report section, and the positive correlation of the two latter for most report sections, indicate that participants were focused on the reading task and read mostly the whole report. In line with this, the exploratory appraisal of pupil dilation showed only a slight variation, which may indicate that cognitive load remained almost constant throughout the reading task. Slight variations of average fixation duration may indicate that information processing seemed to vary in favor of the discussion and conclusion section and at the disadvantage of the methods section. Secondly, it may show how the different types of data relate to each other, which would be rather complementary. The heatmaps gave a fast overview of what sections of the report were fixated, whereas fixational and pupillometric measures may indicate whether these fixations were due to attention and information processing. Interviews provided further information on the reasons why certain areas of the report received more fixations than others. Further, the limited attention and appreciation for methods in a quantitative study should be more intensively studied, as understanding the methods is a condition to assess the quality of the provided information. Here, it would be interesting to investigate further on, for example, the effect of another presentation format of key methods information on the perception methodological information and the interpretation of results. Health policy decisions are complex and comprise many aspects beside research evidence, such as values, which then are expressed in decision criteria. In our decision scenario, no specific values or aims were provided to participants. Hence, none of the three options for long-term care can be regarded as the ‘correct choice’. For future research, predefining such a correct choice could deliver more insight into the role of factors such as risk numeracy. In our study, we did not obtain data about the participants’ knowledge of the decision scenario topic (dementia). Nevertheless, examining the influence of this knowledge on the time reading any given section could be a potential area of further research. Furthermore, we did not collect information, whether German was a second language for any of the participants. The influence of these skills on reading a data report could be another potential area of research.

## Conclusions

This study showed that (future) health decision-makers spend in general equal time and attention to all parts of a presented quantitative data report. However, how much time was spent on the methods section varied considerably among participants and they were less focused while reading this section. Participants’ perceptions of understandability and helpfulness were hardly related to measures based on eye-tracking. Thus, eye-tracking adds additional information that helps to understand the reading of quantitative data reports by health decision-makers.

## Supplementary Information

**Additional file 1.** Quantitative data report.

**Additional file 2.** Questionnaire.

**Additional file 3.** Interview guide.

**Additional file 4.** Heatmaps of all 46 participants.

**Additional file 5.** Graphics: Average fixation duration and time spent per report section.

## Data Availability

The datasets analyzed during the current study are available from the corresponding author on reasonable request.

## Abbreviations

HPM: Health policymaking
IPG: Bachelor program interprofessional healthcare
HITS: Heidelberg Institute for Theoretical Studies
FPS: Frames per second
SD: Standard deviation
