# Supplementary material for: Use of a quantitative data report in a hypothetical decision scenario for health policymaking: a computer-assisted laboratory study
Source: BMC Med Inform Decis Mak. 2021 Jan 28;21:32. doi: 10.1186/s12911-021-01401-4 (PMC7845041; doi:10.1186/s12911-021-01401-4)
Supplement: Supplementary file 1 — Additional file 1. Quantitative data report. [file 12911_2021_1401_MOESM1_ESM.pdf]

Quantitative data report
# - based on hypothetical decision scenario

Original language version (German): p. 2-14
English version (translated by the authors): p. 15-27

Situation der Langzeitpflege im Rhein-Neckar-Kreis

Bericht der Arbeitsgruppe Versorgung im ländlichen Raum der Kommunalen Gesundheitskonferenz des Rhein-Neckar-Kreises

Inhalt

1. Einleitung 4

1.1 Aktuelle Herausforderungen 4

1.2 Zusätzliche Mittel für die Pflege 4

1.3 Ziele der vorliegenden Analyse 5

2. Methodik 5

2.1 Datenquellen 5

2.2 Definitionen 7

2.3 Analysemethoden 8

3. Ergebnisse 9

3.1 Bevölkerungsentwicklung 9

3.2 Pflegeangebote 10

3.3 Nachfrage von Pflegeangeboten 10

4. Diskussion und Schlussfolgerungen 13

1. Einleitung

1.1 Aktuelle Herausforderungen

Der Rhein-Neckar-Kreis möchte seinen Bürgerinnen und Bürgern aktuell und zukünftig eine gute und qualitativ hochwertige Versorgung mit Langzeitpflege anbieten. Unter Langzeitpflege fallen sämtliche Pflegemaßnahmen, die über einen längeren Zeitraum oder auf Dauer erbracht werden. Bei der Kommunalen Gesundheitskonferenz (CHC) 2018 des Rhein-Neckar-Kreises berichteten Pflegebedürftige, ihre Angehörigen, aber auch Mitarbeiter aus ambulanten Pflegediensten und Pflegeheimen von wachsenden Schwierigkeiten, den aktuellen Bedarf an Pflegeleistungen zu decken. Es wurde unter anderem berichtet von steigenden Wartezeiten für einen Platz der vollstationären Dauerpflege, mehr Pflegebedürftigen einerseits und weniger Zeit für die Pflege durch Pflegekräfte. Pflegende Angehörige berichten, dass sie oft stark belastet sind durch die Pflege, sich gezwungen sehen, ihre berufliche Tätigkeit zu reduzieren oder vollständig aufzugeben und oft weniger Zeit haben für die Selbstsorge, z.B. weniger Kontakt zu Freunden haben und stärker gesundheitlich beeinträchtigt sind.

Der bundesweite demografische Wandel steht auch im Rhein-Neckar-Kreis bevor bzw. findet bereits statt und birgt vielfältige Herausforderungen für die Langzeitpflege. Es wird erwartet, dass zukünftig mehr ältere Menschen und damit mehr Pflegebedürftige im Kreis leben werden. Ebenso erwartet wird die Zunahme von Menschen mit Demenz, deren Pflege mit besonderen Anforderungen an Pflegeangebote geknüpft ist. Auf der anderen Seite werden weniger Pflegende erwartet sowohl unter den Angehörigen von Pflegebedürftigen als auch unter Fachkräften. In Deutschland werden derzeit viele Pflegebedürftige durch ihre Kinder gepflegt. Immer mehr Menschen bleiben in ihrem Leben jedoch kinderlos. Ebenfalls übernehmen meist Frauen die Pflege von Angehörigen. Die Zahl der berufstätigen Frauen ist gestiegen, was bedeutet, dass sie weniger Kapazitäten für die Pflege von Angehörigen haben werden. Im Bereich der Fachkräfte haben bereits jetzt immer mehr Einrichtungen Schwierigkeiten, Personal für die Pflege zu gewinnen. Durch den demografischen Wandel wird eine Verschärfung dieser Situation erwartet.

Auch aus den Nachbarregionen des Rhein-Neckar-Kreises werden Schwierigkeiten bei der Vorhaltung bedarfsgerechter Angebote der Langzeitpflege berichtet und auch zukünftig erwartet, sodass Bürgerinnen und Bürger derzeit und in den nächsten Jahren keine Möglichkeit haben, auf Angebote aus benachbarten Regionen auszuweichen.

Die Langzeitpflege steht bundesweit vor den genannten Herausforderungen. Daher wurden bereits einige Maßnahmen umgesetzt, deren Wirksamkeit vor Ort abzuwarten ist. Um dem Fachkräftemangel zu begegnen sollen mit der Beitragssteigerung in der Pflegeversicherung im Jahr 2019 bundesweit 13.000 neue Stellen für Pflegekräfte geschaffen werden, die Pflegeausbildung reformiert und Pflegekräfte besser bezahlt werden. Mit den drei Pflegestärkungsgesetzen (PSG I-III) wurden u.a. Maßnahmen zur Verbesserung des Pflegeschlüssels in Pflegeheimen, unterstützende Maßnahmen für pflegende Angehörige (z.B. kostenlose Beratungsangebote, bessere soziale Absicherung) und mehr Leistungsansprüche für Pflegebedürftige eingeleitet.

1.2 Zusätzliche Mittel für die Pflege

Im Anschluss an die CHC 2018 hat der Landrat des Rhein-Neckar-Kreises angekündigt, zusätzliche finanzielle Mittel für die Langzeitpflege bereitzustellen und möchte bei der Mittelverwendung Empfehlungen der CHC berücksichtigen. Die Mittel sollen von 2020 bis 2030 jährlich ausgezahlt werden. Die

genaue Höhe der Mittel ist noch nicht bekannt. Des Weiteren können die Mittel nur für eine von drei bereits festgelegten Optionen verwendet werden. Diese werden nachfolgend mit einer kurzen Einschätzung ihrer budgetären Konsequenzen vorgestellt.

Option A: Aufstockung von Mitteln für Unterstützungsmaßnahmen pflegender Angehöriger (z.B. Geldleistungen, Beratungsangebote, niedrigschwellige Betreuung, Aufstockung von Plätzen der Kurzzeitpflege in Pflegeheimen). Dies ist die pro Kopf gerechnet kostengünstigste Option. D.h. die meisten Pflegebedürftigen würden von dieser Option profitieren. Professionelle Pflegeleistungen sind von dieser Option ausgeschlossen.

Option B: Aufstockung von Pflegeplätzen der vollstationären Dauerpflege. Diese Option ist pro Kopf gerechnet die teuerste Variante, d.h. im Vergleich zu den anderen Optionen würden die wenigsten Pflegebedürftigen profitieren.

Option C: Aufstockung von Kapazitäten in der ambulanten Pflege. Durch diese Option können ambulante Pflegedienste ihre Kapazitäten erhöhen, um mehr Pflegebedürftige zu versorgen und/oder ihre Leistungen je Pflegebedürftigem auszuweiten, z.B. in Form von mehr Pflegezeit. Diese Option ist pro Kopf gerechnet günstiger als Option B und teurer als Option A, gleich verhält es sich mit der Anzahl der Pflegebedürftigen, die von dieser Option profitieren würden.

1.3 Ziele der vorliegenden Analyse

Die Arbeitsgruppe „Versorgung im ländlichen Raum“ der CHC Rhein-Neckar-Kreis sieht sich verpflichtet, ihren Beitrag für Empfehlungen an den Landrat zur Verwendung der geplanten zusätzlichen Mittel für die Langzeitpflege zu geben. Für eine datengestützte Empfehlung wurde daher der vorliegende Bericht von Mitgliedern der AG erstellt. Ziel des Berichtes ist eine zusammenfassende Darstellung der aktuellen und Schätzung der zukünftigen Versorgungssituation im Bereich der Langzeitpflege im Rhein-Neckar-Kreis anhand verfügbarer Daten. Da das Krankheitsbild Demenz zu besonderen Pflegebedarfen bei Betroffenen führt, sollten hierzu ebenfalls Analysen erstellt werden. Folgende Fragestellungen sollen mit dem vorliegenden Bericht für den Rhein-Neckar-Kreis bearbeitet werden:

- Wie viele Pflegebedürftige (insbesondere mit Demenz) leben derzeit und zukünftig im Kreis?
- Wie ist die derzeitige und zukünftige Inanspruchnahme von ambulanten Pflegediensten und Plätzen der vollstationären Dauerpflege?
- Wie ist die bisherige Entwicklung des Pflegeangebotes und dessen Auslastung?

2. Methodik

Die durchgeführten Analysen basieren zum einen auf Sekundärdatenauswertungen der AG-Mitglieder. Dort, wo keine Daten zur Verfügung standen, wurden Ergebnisse aus anderen Berichten oder Studien für den Ergebnisteil herangezogen.

2.1 Datenquellen

Für die Sekundärdatenanalyse wurden verschiedene Datenquellen ausgewertet, die nachfolgend vorgestellt werden.

Routinedaten der AOK Baden-Württemberg (AOK BW): Daten zur aktuellen und Projektion der Inanspruchnahme von Pflegeleistungen sowie demenzbezogene Analysen basieren auf Abrechnungsdaten der gesetzlichen Kranken- und Pflegekasse AOK BW aus dem Zeitraum 01.01.2013 bis 31.12.2013. In diesem Zeitraum waren rund 28% der Wohnbevölkerung im Rhein-Neckar-Kreis bei der AOK BW versichert. Damit ist die Stichprobe dieser Datenquelle sehr umfassend. Dennoch ist mit historisch entstandenen Unterschieden zwischen der AOK Versichertenpopulation und der Gesamtbevölkerung im Rhein-Neckar-Kreis zu rechnen, die die Repräsentativität der Daten limitieren können. Die Sicherung der Datenqualität gemäß aktueller Standards (Gute Praxis Sekundärdatenanalyse 2012) erfolgte durch das aQua-Institut für angewandte Qualitätsförderung und Forschung im Gesundheitswesen. Im Rahmen der Berichterstellung durch die AG der CHC wurden diese Daten anschließend über sichere Verbindungen an die AG übermittelt. Für die Kodierung der Versichertendiagnosen wurde die Internationale statistische Klassifikation der Krankheiten und verwandter Gesundheitsprobleme in der zehnten Revision nach der deutschen Modifikation (ICD-10-GM) verwendet.

Pflegestatistik: Daten zum Pflegeangebot, zu bisherigen Entwicklungen bei den Pflegebedürftigen und der Inanspruchnahme von Pflegeleistungen durch Pflegebedürftige basieren auf Daten der Pflegestatistik des Statistischen Landesamtes Baden-Württemberg. Die Statistik ist eine Vollerhebung und wird seit 1999 zweijährlich zum Stichtag 15. Dezember eines Jahres erhoben. Zweck der Statistik ist die Erfassung von Angebot und Nachfrage im Bereich der Pflege für die Planung der Versorgung. Der Zugang zu den Daten erfolgte online über die Regionaldatenbank des Statistischen Landesamtes Baden-Württemberg.

Regionalisierte Bevölkerungsvorausrechnung: Projizierte Daten basieren auf der Regionalisierten Bevölkerungsvorausberechnung des Statistischen Landesamtes Baden-Württemberg. Die aktualisierte und im vorliegenden Bericht verwendete Vorausberechnung wurde im Jahr 2016 veröffentlicht und anlässlich des besonders hohen Zuzugs von Flüchtlingen im Jahr 2015 für die Jahre 2014 bis 2060 auf Landesebene und bis zum Jahr 2035 in der regionalisierten Variante erstellt. Berücksichtigte Parameter in der Vorausberechnung beziehen sich auf natürliche Wanderungsbewegungen (Geburten, Sterbefälle), Wanderungsbewegungen innerhalb des Landes und mit dem Ausland. Je nach Parameter wurden unterschiedlich lange Stützzeiträume verwendet, deren Obergrenze das Jahr 2014 bildet. Bis zu diesem Jahr lagen auf dem Zensus 2011 basierende Statistiken zum fortgeschriebenen Bevölkerungsstand vor. Es wurden drei verschiedene Entwicklungskorridore für den Rhein-Neckar-Kreis berechnet, die sich in ihren Annahmen unterscheiden. Der Hauptvariante liegen folgende Annahmen zugrunde:

- Bei der Lebenserwartung der Frauen wird von einer weiteren Zunahme um 2,1 Jahre bis zum Jahr 2035 ausgegangen, bei den Männern leicht höher mit 2,4 Jahren.
- Es wird von einem leichten Anstieg der Geburtenrate ausgegangen von 1,46 auf 1,5. Hierzu wird ferner für die nächsten 10 Jahre angenommen, dass sich der Trend, Kinder später zu bekommen, fortsetzt und dann konstant bleibt.
- Es wird ein Wanderungsgewinn von 125.000 Personen angenommen für das Land Baden-Württemberg, der für jedes Folgejahr abnimmt.

Die drei Varianten der Bevölkerungsvorausrechnung unterscheiden sich nur in den Annahmen zu Fort- und Zuzügen in den Landkreis. In der Hauptvariante wird für den Zeitraum zwischen 2015 bis 2060 ein Wanderungssaldo von 62.870 Personen angenommen, für die untere Variante 47.901 Personen und die obere Variante 97.299 Personen.

2.2 Definitionen

Die Definition der im Bericht betrachteten Pflegeleistungen sowie der Pflegebedürftigkeitsbegriff beruhen auf dem Pflegeversicherungsgesetz (SGB XI).

Pflegebedürftigkeit

Gesetzlich Pflegeversicherte gelten dann als pflegebedürftig, wenn dies gutachterlich festgestellt wurde. Hierzu wird auf Antrag bei der Pflegekasse ein Gutachter bestellt, i.d.R. vom Medizinischen Dienst der Krankenversicherung. Die Begutachtung orientiert sich dabei am gesetzlich festgelegten Pflegebedürftigkeitsbegriff. Bis einschließlich des Jahres 2016 bezog sich die Pflegebedürftigkeit vorrangig auf körperliche Beeinträchtigungen und den damit verbundenen zeitlichen Hilfebedarf bei überwiegend körperlichen Verrichtungen. Je nach Ausmaß des festgestellten Pflegebedarfs erfolgte eine Zuteilung zu einer von drei Pflegestufen und zusätzlich die Feststellung einer „erheblich eingeschränkten Alltagskompetenz", der sogenannten Pflegestufe 0, zum Beispiel aufgrund einer Demenz. Mit dem Pflegestärkungsgesetz wurde zum 1. Januar 2017 ein neuer und weiter gefasster Pflegebedürftigkeitsbegriff eingeführt. Hierüber sollen geistig bzw. psychisch bedingte Beeinträchtigungen stärker als bisher berücksichtigt werden. Die Einstufung des Pflegebedarfs erfolgt in fünf Pflegegraden. Der vorliegende Bericht beruht hauptsächlich auf Daten vor 2017, sodass die Darstellung der Pflegebedürftigkeit hier auf der Definition vor 2017 basiert.

Vollstationäre Dauerpflege

Das Angebot stationärer Pflegeeinrichtungen (Pflegeheime) wird unterschieden zwischen Kurzzeitpflege in einer vollstationären Einrichtung bis zu acht Wochen im Jahr, teilstationärer Pflege in Form von Tages- und/oder Nachtpflege und vollstationärer Dauerpflege. Die Auswertungen in diesem Bericht beziehen sich auf die vollstationäre Dauerpflege.

Ambulante Pflege

Pflegebedürftige, die in häuslicher Umgebung leben, können ambulante Pflegedienste nutzen. Die Leistungen umfassen körperbezogene Pflegemaßnahmen wie waschen, anziehen, beim Essen helfen, pflegerische Betreuungsmaßnahmen sowie Hilfe bei der Haushaltsführung, z.B. einkaufen oder kochen. Diese sogenannten Pflegesachleistungen dürfen nur zugelassene Pflegedienste erbringen, mit denen Pflegekassen einen Versorgungsvertrag abgeschlossen haben.

Demenzen – Definition und Einteilung

Eine Demenz (lat. *dementia* = ohne Verstand) wird derzeit definiert als ein psychopathologisches Syndrom. Dieses setzt sich zusammen aus einer erworbenen Störung von Gedächtnisfunktionen und mindestens einer weiteren kognitiven Beeinträchtigung des Denkens, der Orientierung, der Auffassung, des Rechnens, der Lernfähigkeit, der Sprache und des Urteilsvermögens. Dazu kommen Veränderungen der emotionalen Kontrolle, des Sozialverhaltens und der Motivation. Kognitive Beeinträchtigungen sind bei Demenzen so stark ausgeprägt, dass sie eine Verschlechterung gegenüber einem vormals höheren Leistungsniveau darstellen und Einschränkungen in der Alltagskompetenz mit sich bringen. Von Demenzen abzugrenzen ist das Delir, bei dem kurzfristige kognitive Einbußen auftreten.

Es gibt verschiedene Ursachen für eine Demenz. Je nach Ursache spricht man von einer primären oder sekundären Demenz. Bei der ersteren und häufigeren Form liegt der Schwerpunkt der Erkrankung hauptsächlich im Gehirn. Die häufigste Ursache einer primären Demenz ist die Alzheimer-Krankheit. Bei sekundären Demenzen ist das Gehirn nur mittelbar in das Krankheitsgeschehen einbezogen, der Schwerpunkt der Krankheitsursache wird außerhalb des Gehirns verortet. Zerebrale Durchblutungsstörungen sind die häufigste Ursache einer sekundären Demenz.

Falldefinition für Menschen mit Demenz bei Routinedatenanalysen

Eigene Berechnungen zur Häufigkeit von Demenzerkrankungen basieren auf Abrechnungsdaten der AOK BW aus der ambulanten und stationären medizinischen Versorgung. Diagnosen werden dort mit der Internationalen statistischen Klassifikation der Krankheiten und verwandter Gesundheitsprobleme, 10. Revision, German Modification (ICD-10-GM) dokumentiert. Ein Versicherter wurde dann als Betroffener gezählt, wenn die folgenden Kriterien erfüllt waren:

- Mindestens eine der folgenden Diagnosen nach ICD-10-GM lag vor: F00, F01, F02, F03, F05.1, G30, G31.0, G31.82.
- Die Demenz-Diagnose lag als ambulant gesicherte Diagnose in mindestens zwei Quartalen des Beobachtungsjahres vor
- ODER die Demenz-Diagnose lag als stationäre Hauptdiagnose bei Entlassung vor
- ODER die Demenz-Diagnose lag als stationäre Nebendiagnose in mindestens zwei stationären Aufenthalten vor.

2.3 Analysemethoden

Administrative Prävalenz der Demenz

Bei Prävalenzschätzungen, die auf der Grundlage von Routinedaten durchgeführt wurden, spricht man auch von der administrativen Prävalenz. Damit wird u. a. betont, dass nur diejenigen „Fälle“ erfasst werden, bei denen eine Erkrankung zu einer Inanspruchnahme von Leistungen - im vorliegenden Fall der gesetzlichen Krankenversicherung - führte. Die Population der AOK BW-Versicherten kann von der Struktur der Wohnbevölkerung im Rhein-Neckar-Kreis abweichen. Deshalb wurden Berechnungen zur administrativen Prävalenz der Demenz angepasst an die Alters- und Geschlechtsstruktur der Wohnbevölkerung des Rhein-Neckar-Kreises mittels Daten der Bevölkerungsfortschreibung des Statistischen Landesamtes Baden-Württemberg. Bis auf die Gruppe der 90-Jährigen und höher erfolgte die Altersadjustierung nach Altersjahren. Aufgrund der Datenverfügbarkeit erfolgte diese Anpassung für die Gruppe der 90-Jährigen und älter nicht in Altersjahren sondern als eine Altersgruppe. Die Adjustierung nach weiteren sozioökonomischen Variablen wie dem sozioökonomischen Status, der in Zusammenhang mit gesundheitsrelevanten Aspekten steht, war aufgrund der verfügbaren Daten nicht möglich.

Projektionen

Projektion bedeutet, dass aktuelle Verhältnisse, z.B. Inanspruchnahme- oder Prävalenzraten auf die künftige Bevölkerung übertragen werden. Die einzige sich ändernde Variable ist demnach die Bevölkerungsstruktur. Die Projektion von Prävalenzen und Inanspruchnahmeraten in den vorliegenden Analysen erfolgte in zwei wesentlichen Schritten. In einem ersten Schritt wurden für jede Alters- und Geschlechtsgruppe (Alter nach Jahren bis zum Alter von 89, danach eine Gruppe für die Altersjahre 90+) jeweils Prävalenz- oder Inanspruchnahmeraten auf Grundlage von Routinedaten der AOK BW ermittelt. Anschließend wurden die alters- und geschlechtsspezifischen Raten mit dem Anteil der vorausberechneten Alters- und Geschlechtsgruppe im Rhein-Neckar-Kreis multipliziert. Die Daten zur vorausberechneten Bevölkerungsstruktur wurden vom Statistischen Landesamt Baden-Württemberg bereitgestellt.

3. Ergebnisse

3.1 Bevölkerungsentwicklung

Erste Hinweise für die zukünftige Entwicklung der Zahl der Pflegebedürftigen und formellen sowie informellen Pflegenden kann die Entwicklung bestimmter Altersgruppen im Rhein-Neckar-Kreis (Abbildung 1) geben.

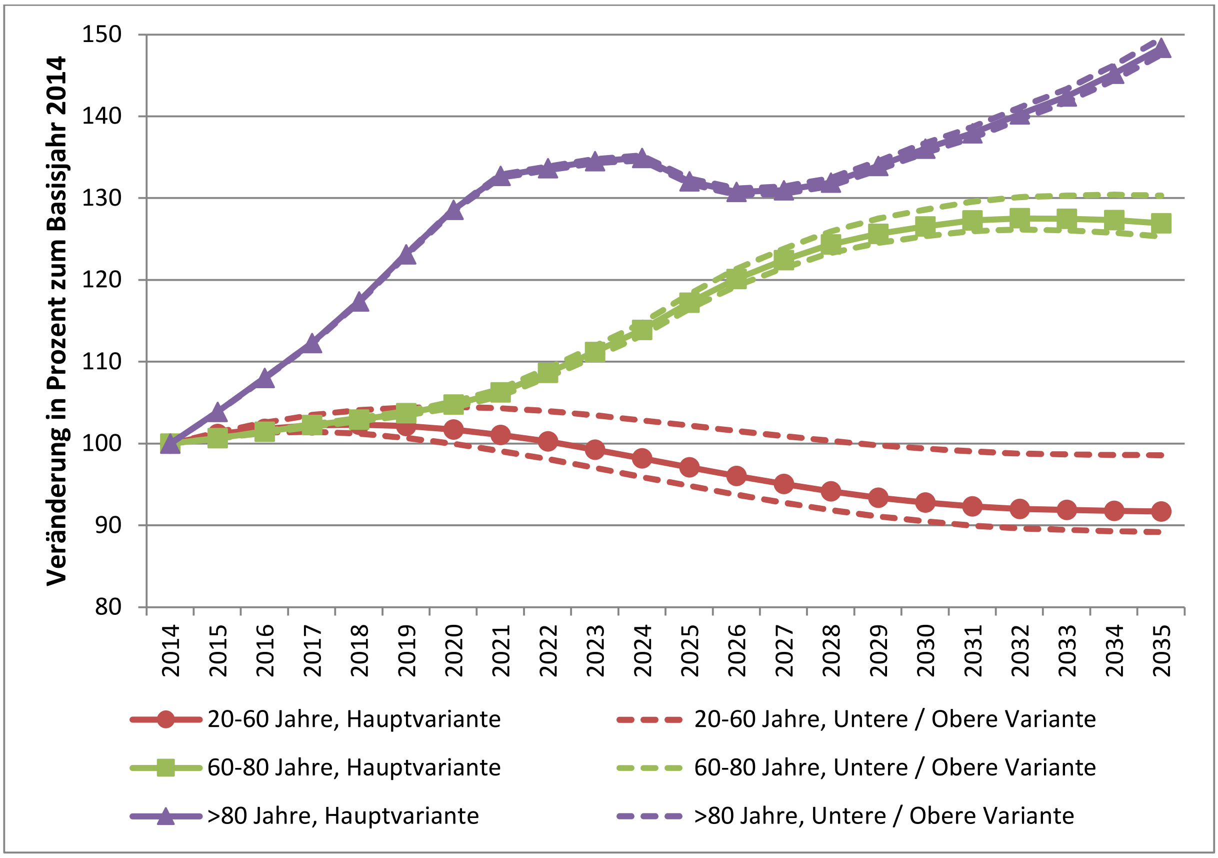

Abbildung 1: Vorausberechnung der Bevölkerungsstruktur im Rhein-Neckar-Kreis nach Altersgruppen von 2014 bis 2035.

In der Altersgruppe der über 80-Jährigen ist das Risiko einer Pflegebedürftigkeit höher als in darunter liegenden Altersgruppen. Ausgehend vom Jahr 2014 wird die Zahl dieser Altersgruppe bis zum Jahr 2035 um fast 50 Prozent zunehmen. Damit ergeben sich für diese Altersgruppe die größten Veränderungen. Anhand der Altersgruppe der 60 bis 80-Jährigen lassen sich erste grobe Schätzungen zur Entwicklung sowohl der Zahl Pflegebedürftiger als auch potenziell pflegender Angehöriger machen. Auch in dieser Gruppe wird zwischen 2014 bis 2035 eine Zunahme erwartet um circa 25 Prozent. In der Altersgruppe der 20 bis 60-Jährigen, die vor allem potenzielle Pflegende umfasst, wird für den Vorausberechnungszeitraum eine Abnahme zwischen ein bis zehn Prozent erwartet.

3.2 Pflegeangebote

Ambulante Pflegedienste

Zwischen 2003 und 2015 nahm die Anzahl der ambulanten Pflegedienste von 48 auf 56 leicht zu. Ebenso wurde ein Anstieg des Personals in den ambulanten Pflegediensten verzeichnet. Kamen im Jahr 2003 im Durchschnitt 21 Beschäftigte auf einen ambulanten Pflegedienst (u.a. Verwaltung, examinierte Gesundheits- und Krankenpflege, Pflegehelfer), waren es 2015 mit 29 etwas mehr. Ein genaueres Maß der absoluten Kapazitäten stellt das Personal ambulanter Pflegedienste in Vollzeitäquivalenten dar. Diese lagen 2013 bei 985. Die Bertelsmann Stiftung geht in ihrer Vorausberechnung für das Jahr 2030 von einem Rückgang auf 931 aus. Basis dieser Vorausberechnung ist die Entwicklung der Bevölkerung im Alter zwischen 20 bis 64 Jahren.

Pflegeheime

Die Zahl der verfügbaren Plätze in Pflegeheimen mit vollstationärer Dauerpflege lag im Jahr 2003 bei 3.900 und hat bis 2015 um 28 Prozent zugenommen. Für die relativen Kapazitäten gemessen in Plätzen der vollstationären Dauerpflege je 1.000 Pflegebedürftige ergibt sich eine Abnahme der Kapazitäten von 328 Plätzen je 1.000 Pflegebedürftige in 2003 auf 267 in 2015. In allen Pflegeheimen insgesamt hat die Zahl des Personals (darin enthalten u.a. Verwaltung, examinierte Alten- und Krankenpflege, Pflegehelfer) im selben Zeitraum um 43 Prozent zugenommen. Die absoluten Kapazitäten der Pflegeheime gemessen in Personal im stationären Pflegedienst in Vollzeitäquivalenten lagen 2013 bei 3.184. Die Bertelsmann Stiftung geht in ihrer Vorausberechnung für das Jahr 2030 von einem Rückgang auf 3.010 aus.

Pflegende Angehörige

Da für den Rhein-Neckar-Kreis keine tiefergehenden Informationen über pflegende Angehörige gefunden werden konnten, werden an dieser Stelle Ergebnisse einer deutschlandweiten Umfrage der gesetzlichen Krankenkasse BARMER, publiziert in ihrem Pflegereport 2018, vorgestellt. Demnach waren zwei Drittel der pflegenden Angehörigen weiblich. Knapp die Hälfte der Hauptpflegepersonen war zwischen 50 und 70 Jahre alt. Ein Drittel der pflegenden Angehörigen gab an, erwerbstätig zu sein, jeder Vierte habe aufgrund der Pflege Arbeit reduziert oder vollständig aufgegeben. Etwa die Hälfte der Hauptpflegepersonen gab an, ihren Partner zu pflegen, danach folgen als Hauptpflegepersonen die Kinder pflegebedürftiger Eltern mit rund 28 Prozent und mit rund zwölf Prozent Eltern, die ihre Kinder pflegen.

3.3 Nachfrage von Pflegeangeboten

Pflegebedürftige

Seit Inkrafttreten der Pflegeversicherung beschreibt die Pflegestatistik eine stetige Zunahme der Zahl der Pflegebedürftigen. Im Rhein-Neckar-Kreis ist diese zwischen 2003 und 2015 um mehr als 50 Prozent gestiegen von rund 11.900 Personen auf 18.700. Bis zum Jahr 2035 ergibt sich ein weiterer deutlicher Anstieg auf rund 26.000 Pflegebedürftige (Abbildung 2). Von den derzeit Pflegebedürftigen sind 64 Prozent weiblich. Im Jahr 2013 konnte erstmals Pflegestufe 0 (erheblich eingeschränkte Alltagskompetenz) vergeben werden. Der Anteil der Pflegebedürftigen in dieser Pflegestufe war am niedrigsten und lag bei vier Prozent. Für die meisten Pflegebedürftigen wurde Pflegestufe 1 vergeben (54 Prozent), gefolgt von Pflegestufe 2 mit 32 Prozent und Pflegestufe 3 mit 10 Prozent.

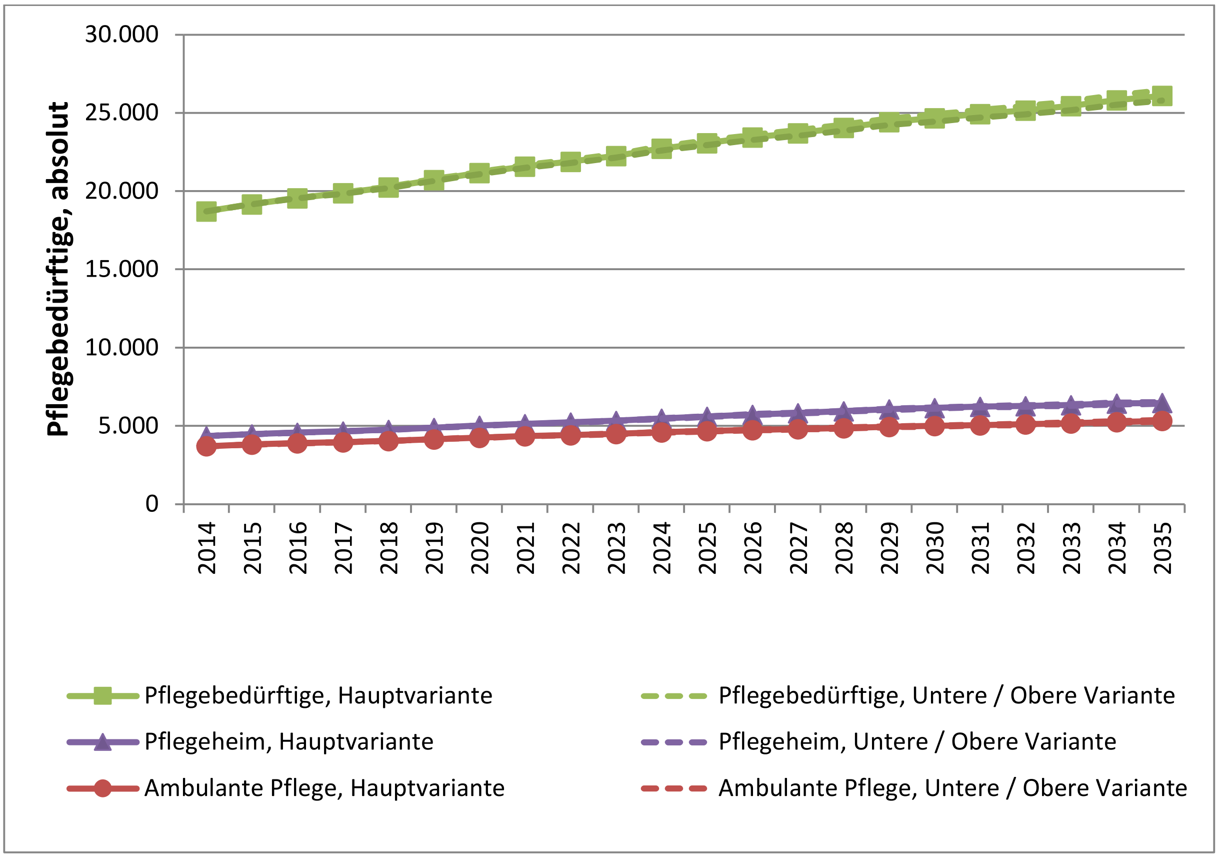

Abbildung 2: Projizierte Entwicklung der Zahl der Pflegebedürftigen und Inanspruchnahme ausgewählter Pflegeangebote im Rhein-Neckar-Kreis von 2014 bis 2035.

Pflege durch Angehörige

Berechnungen der Bertelsmann Stiftung zufolge wurden im Rhein-Neckar-Kreis 53,5 Prozent der Pflegebedürftigen ausschließlich durch Angehörige gepflegt. Für das Jahr 2030 wird ein leichter Rückgang auf 50,7 Prozent erwartet. Einen Einblick in den Pflegeaufwand gibt die BARMER-Versichertenbefragung 2018 für ihre Versicherten in Deutschland insgesamt. Von den befragten pflegenden Angehörigen gaben 85 Prozent an, sich täglich um die pflegebedürftige Person zu kümmern. Die Hälfte davon gab an, sich mehr als zwölf Stunden täglich zu kümmern. Dabei erstreckt sich der Hilfebedarf nicht nur auf Tageszeiten. Etwa zwei Drittel der befragten Hauptpflegepersonen gaben an, sich auch nachts um den Pflegebedürftigen zu kümmern.

Inanspruchnahme ambulanter Pflegedienste

Im Rhein-Neckar-Kreis wurden 2015 rund 20 Prozent der Pflegebedürftigen durch einen ambulanten Pflegedienst versorgt. In den betrachteten Vorjahren (2003 bis 2013) unterlag dieser Anteil leichten Schwankungen, die alle einen niedrigeren Anteil ausweisen mit dem niedrigsten Anteil im Jahr 2005 mit 18 Prozent. Zusammen mit der Zahl der Pflegebedürftigen hat auch die Zahl derjenigen, die einen ambulanten Pflegedienst nutzen, deutlich zugenommen von 2.216 im Jahr 2003 zu 3.715 in 2015. Abbildung 2 zeigt u.a. die projizierte Entwicklung der Zahl der Pflegebedürftigen im Rhein-Neckar-Kreis, die ambulante Pflegedienste in Anspruch nehmen. Demnach würde sich der bisherige Trend der kontinuierlichen Zunahme im projizierten Zeitraum (2014 bis 2035) in allen drei Varianten der

Vorausberechnung fortsetzen. Im Jahr 2035 wären dann circa 5.215 Pflegebedürftige zu erwarten, die durch ambulante Dienste gepflegt würden.

Inanspruchnahme der stationären Pflege

Etwa 23 Prozent der Pflegebedürftigen im Rhein-Neckar-Kreis lebten im Jahr 2015 dauerhaft in einem Pflegeheim und erhielten dort vollstationäre Pflege. Im Vergleich zu den ambulanten Pflegediensten unterlag der Anteil der vollstationären Dauerpflege in den Jahren zwischen 2003 und 2015 größeren Schwankungen. Zunächst war eine kontinuierliche Zunahme zu beobachten, die 2009 einen Höhepunkt fand mit rund 30 Prozent. Danach sank der Anteil kontinuierlich. In absoluten Dimensionen war, wie im Bereich der ambulanten Pflege auch, eine kontinuierliche Zunahme der Pflegebedürftigen in vollstationärer Dauerpflege zu beobachten von 3.340 im Jahr 2003 zu 4.364 in 2015. Auch zukünftig ist mit einer weiteren kontinuierlichen Zunahme zu rechnen (siehe Abbildung 2), die im Jahr 2035 bei etwa 6.383 Pflegebedürftigen in Pflegeheimen läge.

Menschen mit Demenz

Im Rhein-Neckar-Kreis leben derzeit etwa 11.000 Menschen mit einer Demenz. Bis zum Jahr 2035 werden es schätzungsweise 15.000 Betroffene sein (Abbildung 3). Die Schätzungen auf Basis der drei Varianten der Bevölkerungsvorausberechnung liegen dabei nah beieinander.

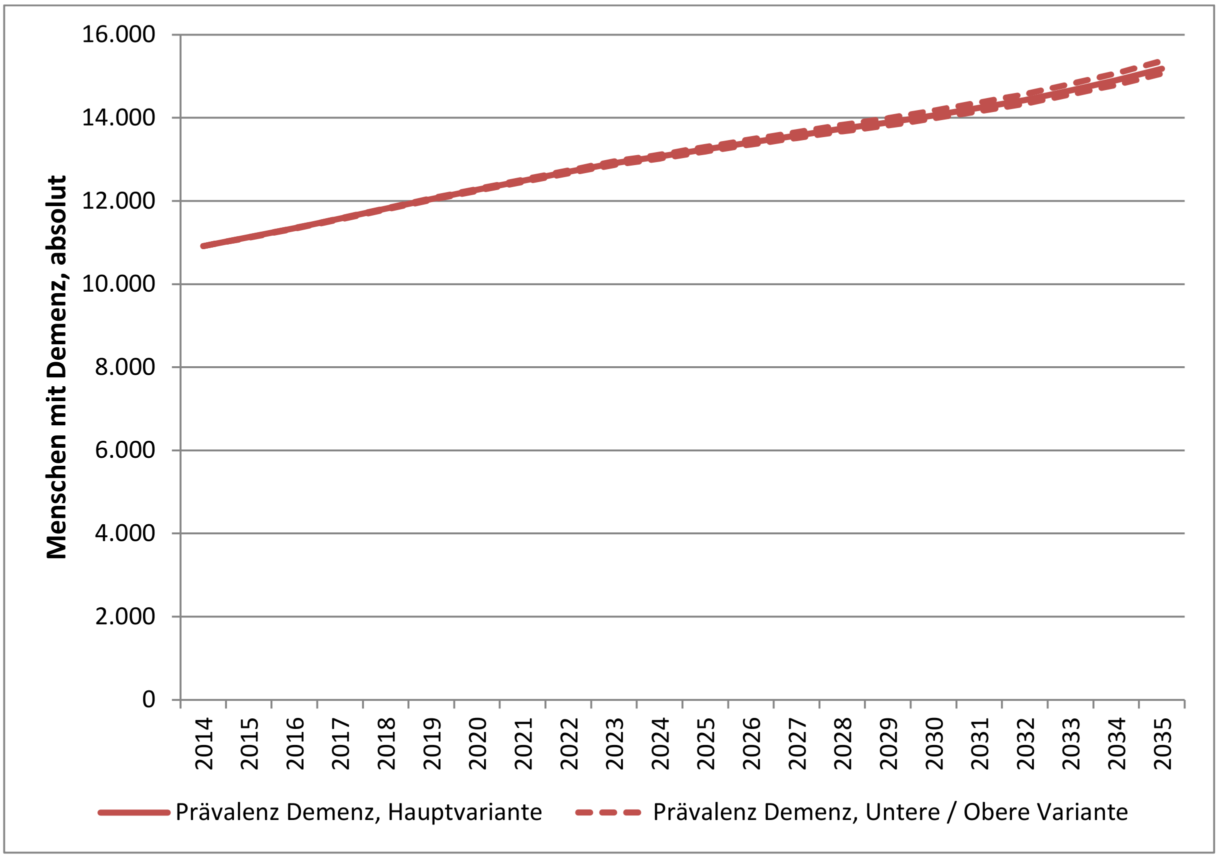

Abbildung 3: Projizierte Entwicklung der Anzahl von Menschen mit Demenz im Rhein-Neckar-Kreis von 2014 bis 2035.

Demenz und Inanspruchnahme von Pflegeleistungen

Umfangreiche Darstellungen zur Inanspruchnahme von Leistungen der gesetzlichen Pflegeversicherung durch Menschen mit Demenz finden sich bei Doblhammer et al. auf Basis von deutschlandweiten

Daten der AOK aus dem Jahr 2007. Die nachfolgenden Ausführungen beziehen sich auf diese Quelle. Das Risiko für Menschen mit Demenz pflegebedürftig zu sein ist groß. Etwa 41 Prozent der an Demenz erkrankten Frauen zwischen 65 und 70 Jahren erhält Pflegeleistungen. Mit dem Alter steigt dieser Anteil kontinuierlich an und stagniert im Alter von 100 Jahren bei circa 93 Prozent. Der Verlauf über das Alter hinweg ist bei Männern ähnlich, ihre Inanspruchnahme unterscheidet sich leicht. So ist der Anteil der Männer mit Demenz, die Pflegeleistungen erhalten, zwischen dem 65. und 75. Lebensjahr mit etwa 47 Prozent höher als bei den Frauen.

Von den pflegebedürftigen Menschen mit Demenz wird ein Großteil durch Pflegeheime versorgt, davon ist die Mehrheit weiblich. Im Alter von 65 Jahren sind die Anteile bei Männern und Frauen noch ähnlich mit jeweils rund 50 Prozent. Danach sinkt der Anteil der im Pflegeheim lebenden pflegebedürftigen Menschen mit Demenz bis zu einem Alter zwischen 75 und 80 Jahren. Bei den Frauen liegt der Anteil hier bei rund 45 Prozent, bei den Männern ist dieser Anteil mit rund 35 Prozent deutlich niedriger. Mit zunehmendem Alter steigt der Anteil bei beiden Geschlechtern und liegt im Alter von 100 Jahren bei mehr als 65 Prozent (Frauen) bzw. rund 50 Prozent (Männer). Eigene Analysen für den Rhein-Neckar-Kreis ergaben, dass circa 40 Prozent der Pflegeheimbewohner über alle Altersgruppen hinweg in vollstationärer Dauerpflege an einer Demenz erkrankt sind. Hoffmann et al. kommen in ihrer Analyse zu dem Schluss, dass für Heimbewohner ab einem Alter von 65 Jahren in Deutschland die Prävalenz der Demenz mit rund 52 Prozent noch höher ist.

4. Diskussion und Schlussfolgerungen

Zusammenfassend zeigen die vorliegenden Analysen, dass für den Rhein-Neckar-Kreis davon auszugehen ist, dass sich bisher beobachtbare Trends sowohl in der Nachfrage als auch im Angebot von Pflegeleistungen fortsetzen werden in den nächsten zehn bis fünfzehn Jahren. Zu berücksichtigen ist, dass die ermittelten Veränderungen aufgrund der Bevölkerungsentwicklung im Kreis zu erwarten ist. Welchen Einfluss aktuelle und zukünftige Maßnahmen wie Gesetzesänderungen und veränderte Lebensweisen haben werden, lässt sich nur schwer abschätzen. Da es sich jedoch um einen vergleichsweise kurzen Zeitraum für die Projektion handelt und die entscheidenden Generationen der Pflegefrage bereits geboren sind, bietet die vorliegende Analyse hilfreiche Informationen über Entwicklungen und ihre Größenordnung von Angebot und Nachfrage von Pflegeleistungen im Kreis.

Die Nachfrage von formeller und informeller Langzeitpflege wird weiterhin zunehmen. Rund 7.000 Pflegebedürftige mehr werden es im Jahr 2035 im Vergleich zu 2014 voraussichtlich sein. Zudem ist zu erwarten, dass unter den Pflegebedürftigen mehr Menschen von einer Demenz betroffen sein werden als bisher. Denn das Risiko an einer Demenz zu erkranken sowie das Risiko für einen Menschen mit Demenz pflegebedürftig zu sein steigt mit dem Alter. Gleichzeitig sind die über 80-Jährigen diejenige Altersgruppe, in der der höchste Bevölkerungszuwachs bis 2035 erwartet wird.

Offen bleibt, wie sich die zunehmende Nachfrage von Pflegeleistungen auf Angehörige, ambulante Pflegedienste und Pflegeheime verteilen. Die Bevölkerungsentwicklung ist hier nur ein Faktor von vielen. Betrachtet man nur die Projektionen aufgrund der Bevölkerungsentwicklung, ergeben sich für die Aufteilung der Pflege kaum Änderungen. Andere Entwicklungen wie die Zunahme von Einpersonenhaushalten und die Tendenz, weniger Kinder zu bekommen, aber auch die Zunahme von Pflegebedürftigen mit höherem Pflegebedarf etwa durch die Zunahme von Pflegebedürftigen mit einer Demenz,

legen nahe, dass die Nachfrage nach formeller Pflege stärker zunehmen könnte als die Nachfrage nach informeller Pflege. Demgegenüber steht der Wunsch vieler Pflegebedürftiger, möglichst lange im häuslichen Umfeld zu verbleiben und durch Angehörige gepflegt zu werden.

Der zunehmenden Nachfrage von Pflegeleistungen steht ein zu erwartender Rückgang der Pflegenden gegenüber. Die Bevölkerungsvorausberechnung liefert hier hilfreiche Informationen. Der vorausberechnete Rückgang des Bevölkerungsanteils von 20 bis 60-Jährigen bedeutet auch einen Rückgang potenzieller professioneller Pflegekräfte. Ob es bis 2035 jedoch zu diesem Rückgang kommt, ist nicht sicher, die Spanne zwischen den drei Vorausberechnungsvarianten reicht hier von einer geringen Abnahme in der oberen Variante bis zu einer Abnahme von rund zehn Prozent gemäß der unteren Variante. Diese Schwankung spiegelt die Unsicherheit über Ab- und Zuwanderung aus bzw. in den Landkreis durch diese Personengruppe wider. Die Entwicklung der formellen Pflegekapazitäten hängt zudem davon ab, wie viele Menschen einen Pflegeberuf als attraktiv ansehen und dementsprechend in diesem Beruf verbleiben bzw. eine Ausbildung beginnen. Geht man nach den gesundheitlichen, beruflichen und finanziellen Belastungen, die pflegende Angehörige derzeit berichten, lässt sich schließen, dass bereits jetzt weitere Unterstützungsangebote zur Entlastung pflegender Angehöriger erforderlich sind. Durch die Zunahme der Pflegebedürftigen insgesamt wird dieser Bedarf in den nächsten Jahren weiter zunehmen.

Situation of long-term care in the Rhine-Neckar district

Report of the Working Group on Care in Rural Areas of the Rhine-Neckar District Communal Health Conference

Table of Contents

1. Introduction 17
   1.1 Current challenges 17
   1.2 Additional funds for long-term care 17
   1.3 Objectives of the present analysis 18
2. Methods 18
   2.2 Definitions 19
   2.3 Methods of analysis 21
3. Results 21
   3.1 Population development 21
3.2 Long-term care supply 22
   3.3 Demand for long-term care services 23
4. Discussion and conclusions 26

1. Introduction

1.1 Current challenges

The Rhine-Neckar district would like to offer its citizens a good and high-quality supply of long-term care currently and in the future. Long-term care includes all care measures that are provided over a longer period of time or on a permanent basis. At the 2018 Communal Health Conference (CHC) of the Rhine-Neckar district, people in need of long-term care, their relatives, and also employees from outpatient care services and nursing homes reported growing difficulties in covering the current demand for long-term care services. Among other things, they reported increasing waiting times for a place in full-time permanent care, more people in need of care on the one hand, and less time for care by caregivers on the other. Family caregivers report that they are often heavily burdened by caregiving, feel forced to reduce or completely abandon their professional activities, and often have less time for self-care, e.g., have less contact with friends and are more impaired in health.

The nationwide demographic change is also imminent or already taking place in the Rhine-Neckar district and poses a variety of challenges for long-term care. It is expected that more elderly people and thus more people in need of care will live in the district in the future. It is also expected that there will be an increase in the number of people with dementia, whose care is linked to special requirements for care services. On the other hand, fewer caregivers are expected, both among the relatives of those in need of care and among professionals. In Germany, many people in need of care are currently cared for by their children. However, more and more people remain childless in their lives. Likewise, it is mostly women who take on the care of relatives. The number of working women has increased, which means that they will have less capacity to care for relatives. In the area of skilled workers, more and more facilities are already having difficulty recruiting personnel for care. Demographic change is expected to exacerbate this situation.

Difficulties in the provision of needs-based long-term care services are also reported from neighboring regions of the Rhine-Neckar district and are also expected in the future so that citizens currently and in the coming years will not have the opportunity to switch to services from neighboring regions.

Long-term care is facing the aforementioned challenges throughout Germany. For this reason, a number of measures have already been implemented, the effectiveness of which remains to be seen on the ground. In order to counter the shortage of skilled workers, the increase in contributions to long-term care insurance in 2019 is to create 13,000 new positions for care workers nationwide, reform care training, and pay care workers better. The three Care Strengthening Acts (PSG I-III) introduced measures to improve the care ratio in nursing homes, support measures for family caregivers (e.g., free counseling services, better social security), and more benefit entitlements for people in need of care.

1.2 Additional funds for long-term care

Following the CHC 2018, the district administrator of the Rhine-Neckar district announced that additional financial resources would be made available for long-term care and would like to take recommendations of the CHC into account when allocating the funds. The funds are to be disbursed annually from 2020 to 2030. The exact amount of the funds is not yet known. Further, the funds can only be used for one of three options already identified. These are presented below with a brief assessment of their budgetary consequences.

Option A: Increase funding for support measures for family caregivers (e.g., cash benefits, counseling services, low-threshold care, increase capacity for short-term care in nursing homes). This is the most cost-effective option on a per capita basis. That is, most people in need of care would benefit from this option. Professional nursing services are excluded from this option.

Option B: Increase nursing home capacity for permanent inpatient care. This option is the most expensive option on a per capita basis, i.e., compared to the other options, the fewest care recipients would benefit.

Option C: Increase capacity in outpatient care. This option allows outpatient care services to increase their capacity to care for more care recipients and/or expand their services per care recipient, e.g., in the form of more care time. This option is cheaper per capita than option B and more expensive than option A, but the same applies to the number of people in need of care who would benefit from this option.

1.3 Objectives of the present analysis

The working group "Healthcare in rural areas" of the CHC Rhine-Neckar district feels obliged to give its contribution for recommendations to the district administrator for the use of the planned additional funds for long-term care. Therefore, the present report was prepared by members of the AG for a data-based recommendation. The aim of the report is to summarize the current and estimate the future supply situation in the field of long-term care in the Rhine-Neckar district on the basis of available data. Since the clinical picture of dementia leads to special care requirements for those affected, analyses should also be prepared for this. The following questions are to be dealt with in this report for the Rhine-Neckar district:

- How many people in need of care (especially with dementia) live in the district at present and in the future?
- What is the current and future utilization of outpatient care services and places of permanent inpatient care in nursing homes?
- What has been the development of long-term care services and their utilization to date?

2. Methods

The analyses carried out are based on the one hand on secondary data analyses by the working group members. Where no data were available, results from other reports or studies were used for the results section.

2.1 Data sources

For the secondary data analysis, various data sources were analyzed, which are presented below.

Routine data of the AOK Baden-Wuerttemberg (AOK BW): Data on the current and projected utilization of long-term care services as well as dementia-related analyses are based on billing data of the statutory health and long-term care insurance company AOK BW from the period 01.01.2013 to 31.12.2013. In this period, about 28% of the resident population in the Rhine-Neckar district were insured with AOK BW. Thus, the sample of this data source is very comprehensive. Nevertheless, historically developed differences between the AOK insured population and the total population in the Rhine-Neckar

district are to be expected, which may limit the representativeness of the data. Data quality assurance according to current standards (Good Practice Secondary Data Analysis 2012) was performed by the aQua-Institute for Applied Quality Promotion and Research in Health Care. As part of the reporting process by the CHC working group, these data were then transmitted to the working group via secure connections. The International Statistical Classification of Diseases and Related Health Problems in the tenth revision according to the German modification (ICD-10-GM) was used for coding the insured diagnoses.

Long-term Care Statistics: Data on the provision of long-term care, previous developments in the number of people in need of care and the utilization of care services are based on data from the Long-term Care Statistics of the Baden-Wuerttemberg State Statistical Office. The statistics are a complete survey and have been collected biennially since 1999 on December 15 of each year. The purpose of the statistics is to record supply and demand in the field of long-term care for the purpose of planning resources. The data is accessed online via the regional database of the Baden-Wuerttemberg Statistical Office.

Regionalized population projection: Projected data are based on the regionalized population projection of the Baden-Wuerttemberg Statistical Office. The updated projection used in this report was published in 2016 and was prepared on the occasion of the particularly high influx of refugees in 2015 for the years 2014 to 2060 at the state level and up to 2035 in the regionalized variant. The parameters taken into account in the projection refer to natural migration (births, deaths), migration within the country and with other countries. Depending on the parameters, support periods of different lengths were used, the upper limit of which is the year 2014. Up to this year, statistics based on the 2011 census were available for the updated population level. Three different development corridors were calculated for the Rhine-Neckar district, which differ in their assumptions. The main variant is based on the following assumptions:

- Life expectancy for women is assumed to increase by a further 2.1 years by 2035, for men slightly higher at 2.4 years.
- A slight increase in the birth rate from 1.46 to 1.5 is assumed. It is also assumed for the next 10 years that the trend toward having children later will continue and then remain constant.
- A migration gain of 125,000 persons is assumed for the state of Baden-Wuerttemberg, which decreases for each subsequent year.

The three variants of the population projection differ only in the assumptions on outward and inward migration to the district. In the main variant, a net migration of 62,870 persons is assumed for the period between 2015 and 2060, 47,901 persons for the lower variant, and 97,299 persons for the upper variant.

2.2 Definitions

The definition of the care services considered in the report and the concept of need for care are based on the German Long-Term Care Insurance Act (SGB XI).

Need for care

People with statutory long-term care insurance are considered to be in need of care if this has been determined by an expert. To this end, an expert is appointed upon application to the long-term care insurance fund, usually by the Medical Service of the Health Insurance Fund. The assessment is based

on the statutory definition of the need for long-term care. Up to and including 2016, the need for care primarily related to physical impairments and the associated need for time-consuming assistance with predominantly physical activities. Depending on the extent of the need for care determined, the person was assigned to one of three care levels and additionally determined to have "significantly limited everyday competence", the so-called care level 0, for example, due to dementia. With the Care Strengthening Act (Pflegestärkungsgesetz), a new and broader definition of the need for care was introduced on January 1, 2017. This is intended to take greater account than before of mental and psychological impairments. The need for care is classified into five degrees of care. This report is mainly based on data prior to 2017, so the presentation of the need for care here is based on the definition prior to 2017.

Full inpatient permanent care

The range of inpatient care facilities (nursing homes) is differentiated between short-term care in a fully inpatient facility for up to eight weeks a year, day care in the form of day and/or night care, and fully inpatient permanent care. The evaluations in this report refer to fully inpatient permanent care.

Outpatient care

People in need of care who live in a home environment can use outpatient care services. The services include body-related care measures such as washing, dressing, helping with meals, nursing care measures, and help with household management, e.g., shopping or cooking. These so-called care benefits in kind may only be provided by licensed care services with which care insurance funds have concluded a care contract.

Dementia - definition and classification

Dementia (lat. dementia = without mind) is currently defined as a psychopathological syndrome. It consists of an acquired disturbance of memory functions and at least one further cognitive impairment of thinking, orientation, perception, calculation, learning ability, language, and judgment. In addition, there are changes in emotional control, social behavior, and motivation. In dementias, cognitive impairments are so pronounced that they represent a deterioration compared to a previously higher level of performance and entail limitations in everyday competence. Delirium, in which short-term cognitive impairment occurs, must be distinguished from dementia.

There are various causes of dementia. Depending on the cause, one speaks of primary or secondary dementia. In the former and more common form, the focus of the disease is mainly in the brain. The most common cause of primary dementia is Alzheimer's disease. In secondary dementias, the brain is only indirectly involved in the disease process, the focus of the disease cause is located outside the brain. Cerebral circulatory disorders are the most common cause of secondary dementia.

Case definition for people with dementia in routine data analyses.

Own calculations on the frequency of dementia are based on administrative data of the AOK BW from outpatient and inpatient medical care. Diagnoses are documented there using the International Statistical Classification of Diseases and Related Health Problems, 10th Revision, German Modification (ICD-10-GM). An insured was counted as a person with dementia if the following criteria were met:

At least one of the following ICD-10-GM diagnoses was present: F00, F01, F02, F03, F05.1, G30, G31.0, G31.82.

- The dementia diagnosis was documented as a confirmed outpatient diagnosis in at least two quarters of the observation year,
- OR the dementia diagnosis was documented as an inpatient principal diagnosis at discharge,
- OR the dementia diagnosis was documented as an inpatient secondary diagnosis in at least two inpatient stays.

2.3 Methods of analysis

Administrative prevalence of dementia
Prevalence estimates based on routine data are also referred to as administrative prevalence. This emphasizes, that only those "cases" are recorded in which a disease led to a claim for services - in the present case, statutory health insurance. The population of AOK BW insurants may differ from the structure of the resident population in the Rhine-Neckar district. Therefore, calculations on the administrative prevalence of dementia were adjusted to the age and gender structure of the resident population of the Rhine-Neckar district using data from the population update of the Baden-Wuerttemberg Statistical Office. Except for the group of 90-year-olds and above, the age adjustment was made according to age years. Due to data availability, this adjustment for the group of 90-year-olds and older was not made in age years but as an age group. Adjustment for other socioeconomic variables, such as socioeconomic status, which is related to health-related aspects, was not possible due to available data.

Projections
Projection means that current conditions, such as utilization or prevalence rates, are applied to the future population. Thus, the only variable that changes is the population structure. The projection of prevalence and utilization rates in the present analyses was done in two main steps. In a first step, prevalence or utilization rates were determined for each age and sex group (age by years up to age 89, then a group for ages 90+) based on routine data from the AOK BW. Subsequently, the age- and sex-specific rates were multiplied by the proportion of the predicted age and sex group in the Rhine-Neckar district. The data on the projected population structure were provided by the Baden-Wuerttemberg State Statistical Office.

3. Results

3.1 Population development

The development of certain age groups in the Rhine-Neckar district (Figure 1) provides initial indications for the future development of the number of people in need of care and formal as well as informal caregivers.

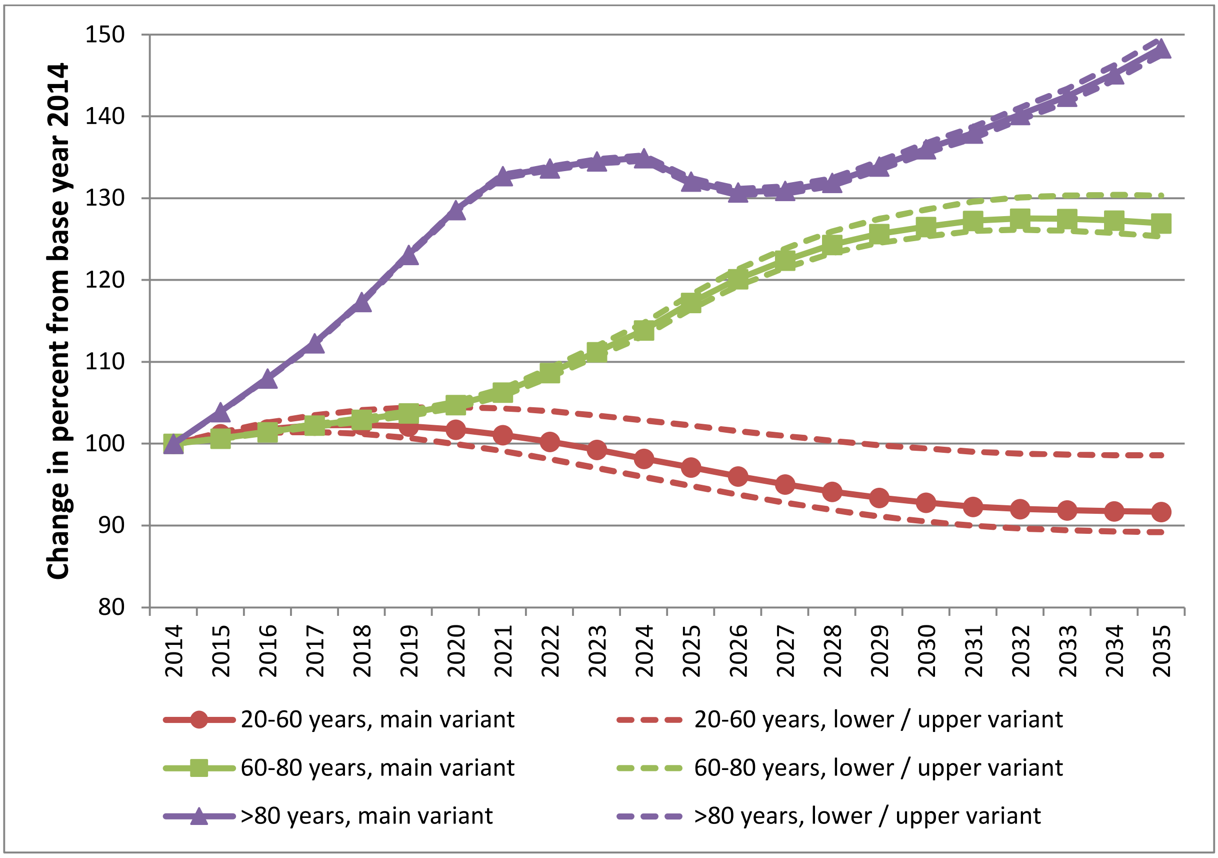

Figure 1: Projection of the population structure in the Rhine-Neckar district according to age groups from 2014 to 2035.

The risk of needing long-term care is higher in the over-80 age group than in the age groups below. Starting from 2014, the number of people in this age group is expected to increase by almost 50 percent by 2035. This means that the greatest changes will occur in this age group. The 60 to 80 age group provides an initial estimate of the development of both the number of people in need of care and the potential number of informal caregivers. In this group, too, an increase of around 25 percent is expected between 2014 and 2035. In the 20 to 60 age group, which mainly comprises potential formal and informal caregivers, a decrease of between one and ten percent is expected for the projection period.

3.2 Long-term care supply

Outpatient care services

Between 2003 and 2015, the number of outpatient care services increased slightly from 48 to 56. Likewise, there was an increase in the number of staff in outpatient care services. Whereas in 2003 there was an average of 21 employees per outpatient care service (including administration, registered health and nursing care, nursing assistants), in 2015 the figure was slightly higher at 29. A more accurate measure of absolute capacity is the number of full-time equivalent staff in outpatient care services. In its projection for 2030, the Bertelsmann Stiftung assumes a decline to 931. This projection is based on the development of the population aged between 20 and 64.

Nursing homes

The number of available places in nursing homes with full inpatient permanent care was 3,900 in 2003 and had increased by 28 percent by 2015. For the relative capacities measured in places of full inpatient permanent care per 1,000 persons in need of care, there is a decrease in capacities from 328 places per 1,000 persons in need of care in 2003 to 267 in 2015. In all nursing homes as a whole, the number of staff (including, among others, administration, registered geriatric nurses, nursing assistants) has increased by 43 percent in the same period. The absolute capacity of nursing homes, measured in full-time equivalent inpatient nursing staff, was 3,184 in 2013. In its projection for 2030, the Bertelsmann Stiftung assumes a decline to 3,010.

Informal caregivers

Since no more in-depth information on caring dependents could be found for the Rhine-Neckar district, results of a Germany-wide survey by the statutory health insurance company BARMER, published in its Long-Term Care Report 2018, are presented here. According to the survey, two-thirds of informal caregivers were female. Just under half of the main caregivers were between 50 and 70 years old. One-third of informal caregivers reported being employed, and one in four had reduced or completely given up work due to caregiving. Around half of the main caregivers stated that they cared for their partner, followed by the children of parents in need of care as the main caregivers with around 28 percent and parents caring for their children with around 12 percent.

3.3 Demand for long-term care services

Persons in need of care

Since long-term care insurance came into force, the long-term care statistics describe a steady increase in the number of persons in need of long-term care. In the Rhine-Neckar district, this number has increased by more than 50 percent between 2003 and 2015, from around 11,900 people to 18,700. By 2035, there will be a further significant increase to around 26,000 persons in need of care (Figure 2). Of those currently in need of care, 64 percent are female. In 2013, care level 0 (significantly impaired daily living skills) was awarded for the first time. The proportion of persons in need of care in this care level was the lowest, at four percent. Care level 1 was awarded to most care recipients (54 percent), followed by care level 2 with 32 percent and care level 3 with 10 percent.

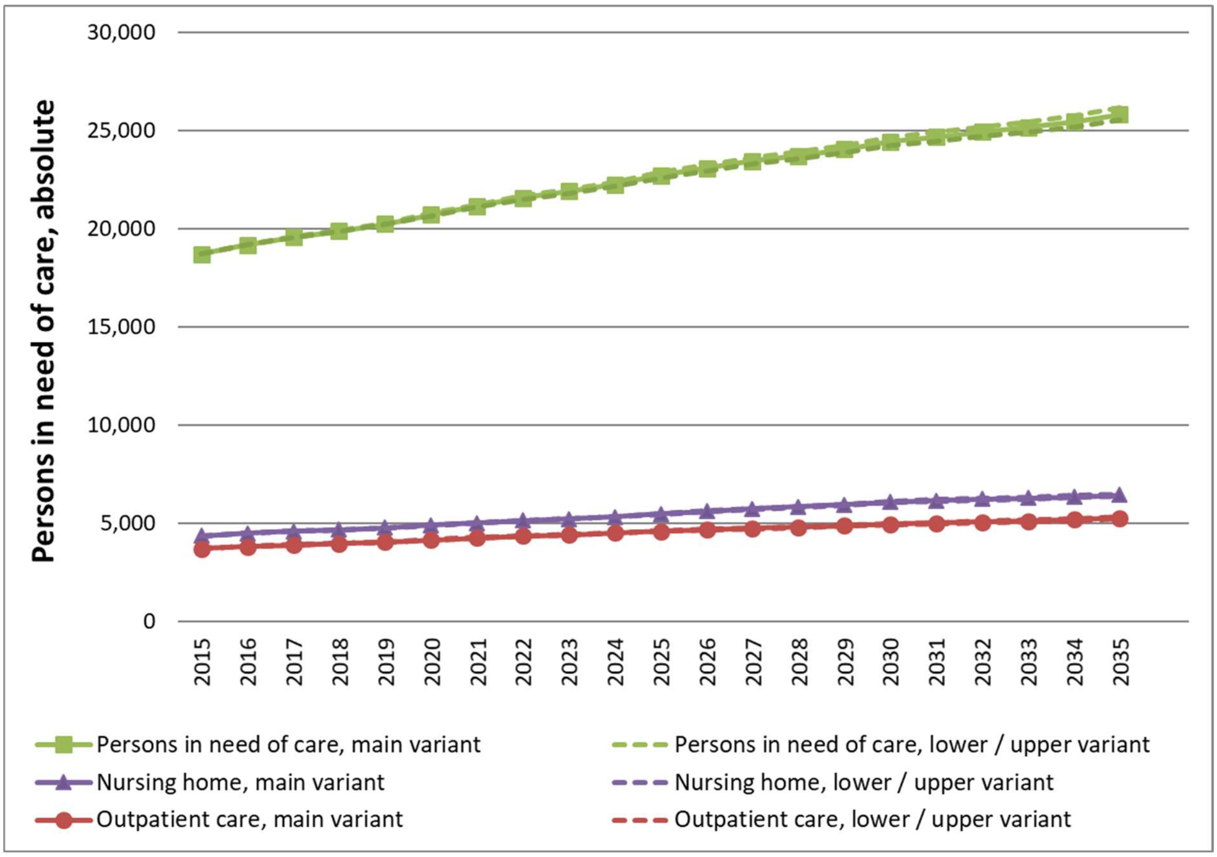

Figure 2: Projected development of the number of persons in need of long-term care and utilization of selected long-term care services in the Rhine-Neckar district from 2014 to 2035.

Care by dependents

According to calculations by the Bertelsmann Stiftung, 53.5 percent of those in need of care in the Rhine-Neckar district were cared for exclusively by their dependents. A slight decline to 50.7 percent is expected for 2030. An insight into the amount of care required is provided by the 2018 BARMER survey of insured persons in Germany as a whole. Of the informal caregivers surveyed, 85 percent said they cared for the person in need of care on a daily basis. Half of them said they cared for more than twelve hours a day. The need for help does not only extend to daytime. Around two-thirds of the main caregivers surveyed also said they looked after the person in need of care at night.

Use of outpatient care services

In the Rhine-Neckar district, around 20 percent of those in need of care were cared for by an outpatient care service in 2015. In the previous years under review (2003 to 2013), this share was subject to slight fluctuations, all of which showed a lower share with the lowest share in 2005 at 18 percent. Along with the number of persons in need of care, the number of those using an outpatient care service has also increased significantly from 2,216 in 2003 to 3,715 in 2015. Figure 2 shows, among other things, the projected development of the number of persons in need of care in the Rhine-Neckar district who use outpatient care services. According to this, the previous trend of continuous increase would continue in the projected period (2014 to 2035) in all three variants of the projection. In 2035, approximately 5,215 people in need of long-term care would then be expected to be cared for by outpatient services.

Use of inpatient care

In 2015, around 23 percent of those in need of care in the Rhine-Neckar district lived permanently in a nursing home and received full inpatient care there. Compared to outpatient care services, the share of full-time inpatient permanent care was subject to greater fluctuations in the years between 2003 and 2015. Initially, there was a continuous increase, which peaked in 2009 at around 30 percent. Thereafter, the share declined continuously. In absolute terms, as in the area of outpatient care, there was a continuous increase in the number of people in need of long-term inpatient care, from 3,340 in 2003 to 4,364 in 2015. A further continuous increase can be expected in the future (see Figure 2), which would amount to around 6,383 people in need of long-term care in nursing homes in 2035.

Persons with dementia

There are currently about 11,000 people living with dementia in the Rhine-Neckar district. By 2035, there will be an estimated 15,000 affected persons (Figure 3). The estimates based on the three variants of the population projection are close to each other.

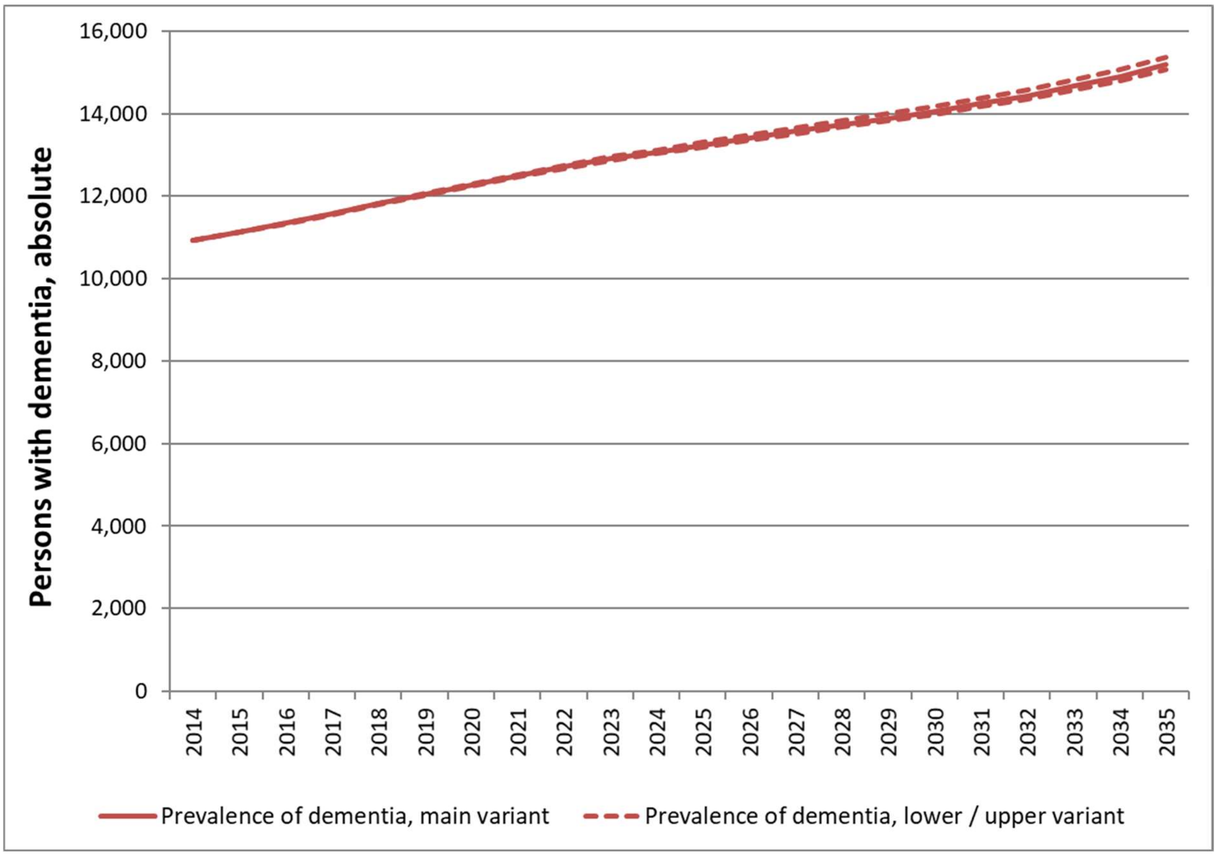

Figure 3: Projected development of the number of persons with dementia in the Rhine-Neckar district from 2014 to 2035.

Dementia and utilization of care services

Doblhammer et al. provide extensive information on the use of long-term care services by persons with dementia, based on nationwide data from the AOK in 2007. The following comments refer to this source. The risk for persons with dementia to be in need of care is high. About 41 percent of women

between the ages of 65 and 70 who are affected by dementia receive care services. This proportion increases steadily with age and stagnates at around 93 percent at age 100. The trend across age is similar for men, but their utilization differs slightly. The proportion of men with dementia receiving care between the ages of 65 and 75 is higher than for women, at around 47 percent.

Of the people with dementia who need care, a large proportion are cared for by nursing homes, the majority of whom are female. At age 65, the proportions are still similar for men and women, at about 50 percent each. Thereafter, the proportion of people with dementia in need of care living in a nursing home decreases until they are between 75 and 80 years old. For women, the proportion here is around 45 percent; for men, the proportion is significantly lower at around 35 percent. With increasing age, the proportion rises for both sexes and is more than 65 percent (women) and around 50 percent (men) at the age of 100. Our own analyses for the Rhine-Neckar district showed that about 40 percent of nursing home residents across all age groups in full inpatient permanent care suffer from dementia. In their analysis, Hoffmann et al. conclude that the prevalence of dementia is even higher for nursing home residents aged 65 and older in Germany, at around 52 percent.

4. Discussion and conclusions

In summary, the present analyses show that for the Rhine-Neckar district, it can be assumed that previously observable trends in both the demand and the supply of care services will continue over the next ten to fifteen years. It must be taken into account that the changes determined are to be expected due to the population development in the district. It is difficult to estimate what influence current and future measures such as changes in legislation and changing lifestyles will have. However, since it is a comparatively short period of time for the projection and the decisive generations of the care issue have already been born, the present analysis provides helpful information about developments and their magnitude of supply and demand of care services in the district.

The demand for formal and informal long-term care will continue to grow. About 7,000 more people in need of long-term care are expected in 2035 compared to 2014. In addition, it is expected that more people will be affected by dementia among those in need of long-term care than in the past. This is because the risk of developing dementia and the risk of a person with dementia needing care increases with age. At the same time, the over-80s are the age group in which the highest population growth is expected by 2035.

It remains to be seen how the increasing demand for care services will be distributed among relatives, outpatient care services and nursing homes. Population development is only one factor of many, here. If we look only at the projections based on population development, there are hardly any changes in the distribution of care. Other developments, such as the increase in single-person households and the tendency to have fewer children, but also the increase in people in need of care with higher care requirements, for example due to the increase in people in need of care with dementia, suggest that the demand for formal care could increase more than the demand for informal care. This contrasts with the desire of many people in need of care to remain in their home environment for as long as possible and to be cared for by relatives.

The increasing demand for care services is offset by an expected decline in the number of people needing care. The population projection provides helpful information here. The predicted decline in the proportion of the population aged 20 to 60 also means a decline in potential professional caregivers.

However, it is not certain whether this decline will occur by 2035; the range between the three projection variants here is from a small decrease in the upper variant to a decrease of around ten percent according to the lower variant. This fluctuation reflects the uncertainty about the outflow and inflow of this group of persons to and from the district. The development of formal nursing capacities also depends on how many people regard a nursing profession as attractive and accordingly remain in this profession or start training. Judging by the health, professional, and financial burdens that caregivers currently report, it can be concluded that further support services are already needed to ease the burden on caregiving dependents. Due to the overall increase in the number of persons in need of care, this need will continue to grow in the coming years.
