# Supplementary material for: Use of a quantitative data report in a hypothetical decision scenario for health policymaking: a computer-assisted laboratory study
Source: BMC Med Inform Decis Mak. 2021 Jan 28;21:32. doi: 10.1186/s12911-021-01401-4 (PMC7845041; doi:10.1186/s12911-021-01401-4)
Supplement: Supplementary file 2 — Additional file 2. Questionnaire. [file 12911_2021_1401_MOESM2_ESM.docx]

**Additional file 2: Questionnaire (used in computer-assisted data collection)**

Note:

This questionnaire was developed for this study and has not been published elsewhere. It consists of validated and non-validated questions. The original language of the questionnaire was German and was translated into English language through one-way translation.

Questions 1–12 and 19–20:

- Non-validated questions from the authors.
- Questions were translated from German into English by the authors.

Questions 13.1–13.8:

- Source: Dalbert C. Die Ungewißheitstoleranzskala: Skaleneigenschaften und Validierungsbefunde. Hallesche Berichte zur Pädagogischen Psychologie. 1999;1.
- Source questions were originally found and validated in German language and therefore were translated from German into English by the authors.

Questions 14–18:

- The question type of the items was adopted from Galesic et al. who tested general graph literacy. In this study questions 14 to 18 were used to measure participants’ adequate understanding of the information presented by graphs in the quantitative data report.
- Source: Galesic M, Garcia-Retamero R. Graph literacy: a cross-cultural comparison. Med Decis Making. 2011;31(3):444-57.
- Questions were translated from German into English by the authors.

Questions 21–24:

- Source: Cokely ET, Galesic M, Schulz E, Ghazal S, Garcia-Retamero R. Measuring Risk Literacy: The Berlin Numeracy Test. Judgment and Decision Making. 2012;7(1):25-47.
- For data collection the German version of items was used.
- In this document (additional file 2) the English version of items is presented.

**Part 1 – At the beginning of data collection**

1. **Please state your year of birth.**

*Please enter your answer here:*

1. **What is your gender?**

*Please select only one of the following answers:*

- male
- female
- other
- no answer

1. **Are you using a visual aid at this moment to work on the study part on the computer screen? (e.g. glasses, contact lenses)**

*Please select only one of the following answers:*

- yes
- no
- no answer

1. **Which visual aid are you using at this moment to work on the study part on the computer screen?**

*Answer this question only if the following conditions are met: Answer was 'yes' to question '3' (Are you using a visual aid at this moment to work on the study part on the computer screen? (e.g. glasses, contact lenses))*

*Please select only one of the following answers:*

- *glasses*
- *contact lenses*
- *other*

1. **How many diopters does your vision aid have?**

*Answer this question only if the following conditions are met: Answer was 'yes' to question '3' (Are you using a visual aid at this moment to work on the study part on the computer screen? (e.g. glasses, contact lenses))*

*If you are not sure about the diopter values, we ask you to estimate which values could be the minimum. If you are still unable to find an answer or if this question does not apply to you (e.g. due to a visual aid that is not designed to compensate for diopters) please enter "0" in the answer field.*

*Only numbers may be entered in these fields.*

*Please enter your answer here:*

*Diopters left eye*

*Diopters right eye*

1. **What material is the lens of your visual aid made of?**

*Answer this question only if the following conditions are met:*

*Answer was 'contact lenses' in question '4 ' (Which visual aid are you using at this moment to work on the study part on the computer screen?)*

*Please select only one of the following answers:*

- glass
- plastic
- don’t know
- no answer
- other

1. **Are your contact lenses...**

*Answer this question only if the following conditions are met:*

*Answer was 'plastic' in question '6 ' (What material is the lens of your visual aid made of?)*

*Please select only one of the following answers:*

- hard
- soft
- don’t know
- no answer

1. **Do you have a university/****non-university degree?**

*Please select only one of the following answers:*

- yes
- no
- no answer

1. **What is the highest university/non-university degree that you have attained?**

*Answer this question only if the following conditions are met:*

*Answer was 'yes' in question '8 ' (Do you have a university/non-university?)*

*Please select only one of the following answers:*

- bachelor’s degree
- master, master’s degree, state examination, teaching examination
- doctoral degree
- habilitation
- other

1. **What is the field of study of your highest university/non-university degree?**

*Answer this question only if the following conditions are met:*

*Answer was 'yes' in question '8 ' (Do you have a university/non-university?)*

*Please select all applicable answers:*

- health sciences
- human medicine/dentistry
- natural sciences
- humanities
- social sciences
- economic sciences
- other

1. **Have you ever dealt with healthcare in practice?**

*(e.g. professional activity, vocational training, part-time work e.g. as a research assistant, internship, civilian service, honorary office, voluntary social year)*

*Please select only one of the following answers:*

- no
- yes:

Please write a comment to your selection

1. **If you summarize all activities, how long was that approximately?**

*Answer this question only if the following conditions are met:*

*Answer was 'yes' in question '11 ' (Have you ever dealt with healthcare in practice?)*

*Please enter your answer here:*

years

If you want to enter month details, please enter them as decimal numbers. Example: 3 months

correspond to 0.25.

*Please mark, which of the following statements apply to you. If you are unsure, please select the answer that applies most.*

|  | is completely not true | is largely not true | is unlikely to be true | is a little bit true | is largely true | is true exactly | no answer |
| --- | --- | --- | --- | --- | --- | --- | --- |
| **13.1 I like to try things out, even if something doesn't always come out of it.** | **⭘** | **⭘** | **⭘** | **⭘** | **⭘** | **⭘** | **⭘** |
| **13.2 I only deal with tasks that can be solved.** | **⭘** | **⭘** | **⭘** | **⭘** | **⭘** | **⭘** | **⭘** |
| **13.3 I like it when unexpected surprises**  **occur.** | **⭘** | **⭘** | **⭘** | **⭘** | **⭘** | **⭘** | **⭘** |
| **13.4 I like to let things slide.** | **⭘** | **⭘** | **⭘** | **⭘** | **⭘** | **⭘** | **⭘** |
| **13.5 I like the work to be even.** | **⭘** | **⭘** | **⭘** | **⭘** | **⭘** | **⭘** | **⭘** |
| **13.6 I'm just waiting for something exciting to happen.** | **⭘** | **⭘** | **⭘** | **⭘** | **⭘** | **⭘** | **⭘** |
| **13.7 When everything goes haywire around me, I feel really good.** | **⭘** | **⭘** | **⭘** | **⭘** | **⭘** | **⭘** | **⭘** |
| **13.8 I like to know what's in store for me.** | **⭘** | **⭘** | **⭘** | **⭘** | **⭘** | **⭘** | **⭘** |

**Part 2 – After decision and reading task**


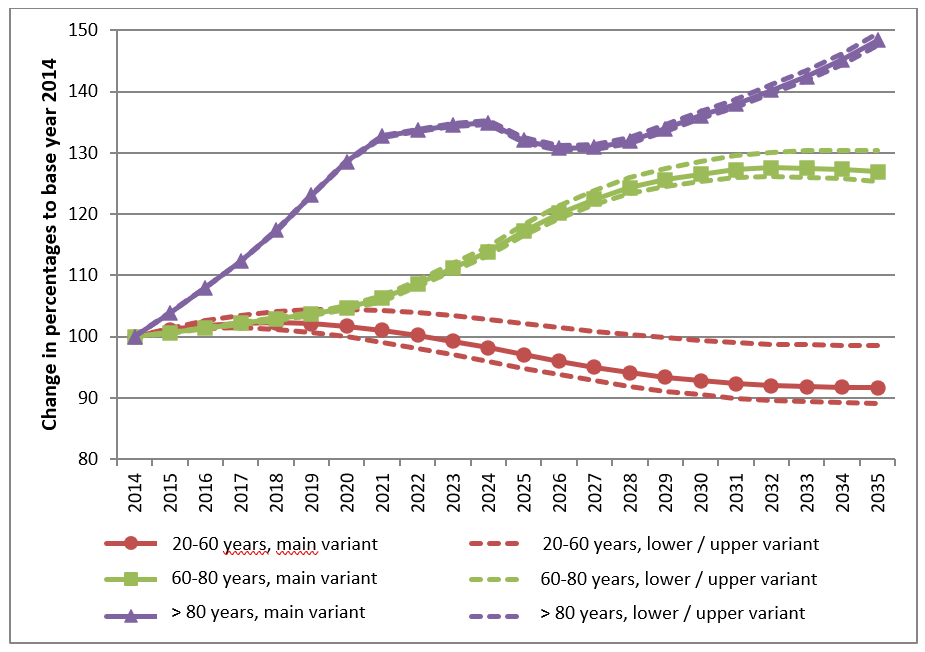


*Figure 1^[[1]](#footnote-1)^: Projection of the population structure in the Rhine-Neckar district according to age groups from 2014 to 2035.*

1. **Compared to the index year 2014 - by how many percent lower/higher is the number of people aged 20-60 expected to be in 2022?**

*Only numbers may be entered in this field.*

*Please enter your answer here:*

1. **In which period is the increase of the population aged 60-80 years expected to be higher?**

*Please select only one of the following answers:*

- from 2014 to 2021
- from 2021 to 2027
- increase is equal in both periods
- don’t know
- no answer

1. **In which age group is the uncertainty of population development highest?**

*Please select only one of the following answers:*

- 20 – 60 years
- 60 – 80 years
- > 80 years
- all equal
- don’t know
- no answer

1. **According to your best estimate - by how many percent lower/higher is the number of people aged 20-60 expected to be in 2038 compared to the index year 2014?**

*Please enter your answer here:*


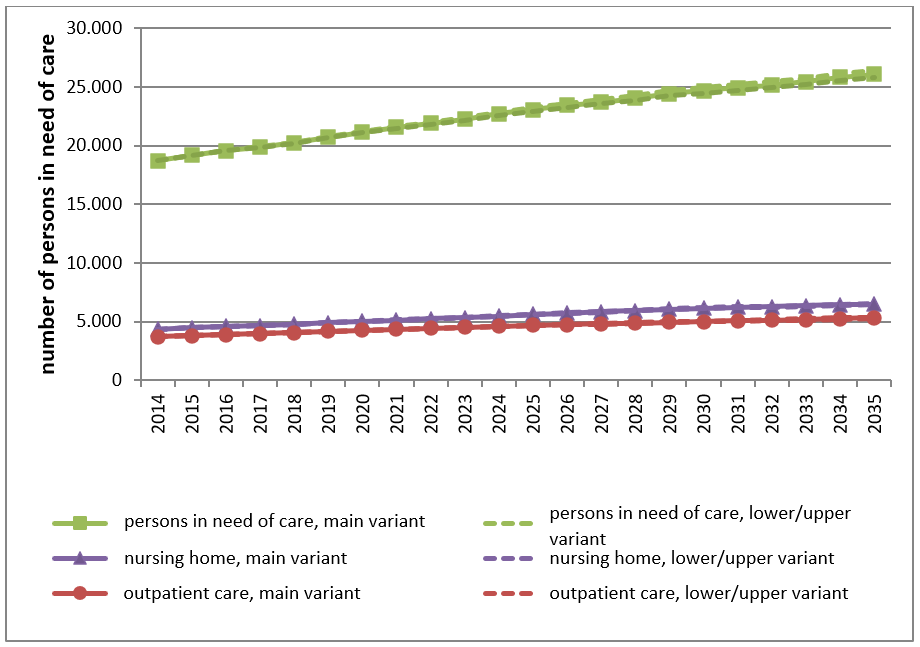


*Figure 2^[[2]](#footnote-2)^: Projected development of the number of persons in need of long-term care and use of selected care services in the Rhine-Neckar district from 2014 to 2035.*

1. **In the period between 2014 and 2035, is it likely that the number of persons using outpatient care or the number of persons in need of care will increase more strongly?**

*Please select only one of the following answers:*

- outpatient care
- persons in need of care
- the number increases at the same rate
- don’t know
- no answer

*The following questions refer to the data report "Situation of long-term care in the Rhine-Neckar district", which you read in the previous task.*

1. **Overall, how helpful did you find the following parts of the report in completing the task?**

*Please select the appropriate answer for each item:*

|  | not helpful at all  1 | 2 | 3 | 4 | 5 | 6 | 7 | 8 | 9 | very helpful  10 | no answer |
| --- | --- | --- | --- | --- | --- | --- | --- | --- | --- | --- | --- |
| **1. Introduction** | **⭘** | **⭘** | **⭘** | **⭘** | **⭘** | **⭘** | **⭘** | **⭘** | **⭘** | **⭘** | **⭘** |
| **2. Methods** | **⭘** | **⭘** | **⭘** | **⭘** | **⭘** | **⭘** | **⭘** | **⭘** | **⭘** | **⭘** | **⭘** |
| **3. Results** | **⭘** | **⭘** | **⭘** | **⭘** | **⭘** | **⭘** | **⭘** | **⭘** | **⭘** | **⭘** | **⭘** |
| **3.1 Results:**  **Figure 1: Projection of the population structure in the Rhine-Neckar district according to age groups from 2014 to 2035** | **⭘** | **⭘** | **⭘** | **⭘** | **⭘** | **⭘** | **⭘** | **⭘** | **⭘** | **⭘** | **⭘** |
| **3.2 Results:**  **Figure 2: Projected development of the number of persons in need of long-term care and use of selected care services in the Rhine-Neckar district from 2014 to 2035** | **⭘** | **⭘** | **⭘** | **⭘** | **⭘** | **⭘** | **⭘** | **⭘** | **⭘** | **⭘** | **⭘** |
| **3.3 Results:**  **Figure 3: Projected development of the number of people with dementia in the Rhine-Neckar district from 2014 to 2035** | **⭘** | **⭘** | **⭘** | **⭘** | **⭘** | **⭘** | **⭘** | **⭘** | **⭘** | **⭘** | **⭘** |
| **3.4 Results: Textual description** | **⭘** | **⭘** | **⭘** | **⭘** | **⭘** | **⭘** | **⭘** | **⭘** | **⭘** | **⭘** | **⭘** |
| **4. Discussion and conclusions** | **⭘** | **⭘** | **⭘** | **⭘** | **⭘** | **⭘** | **⭘** | **⭘** | **⭘** | **⭘** | **⭘** |

*The following questions refer to the data report "Situation of long-term care in the Rhine-Neckar district", which you read in the previous task.*

1. **Overall, how understandable did you find the following parts of the report?**

*Please select the appropriate answer for each item:*

|  | not understandable at all  1 | 2 | 3 | 4 | 5 | 6 | 7 | 8 | 9 | very understandable  10 | no answer |
| --- | --- | --- | --- | --- | --- | --- | --- | --- | --- | --- | --- |
| **1. Introduction** | **⭘** | **⭘** | **⭘** | **⭘** | **⭘** | **⭘** | **⭘** | **⭘** | **⭘** | **⭘** | **⭘** |
| **2. Methods** | **⭘** | **⭘** | **⭘** | **⭘** | **⭘** | **⭘** | **⭘** | **⭘** | **⭘** | **⭘** | **⭘** |
| **3. Results** | **⭘** | **⭘** | **⭘** | **⭘** | **⭘** | **⭘** | **⭘** | **⭘** | **⭘** | **⭘** | **⭘** |
| **3.1 Results:**  **Figure 1: Projection of the population structure in the Rhine-Neckar district according to age groups from 2014 to 2035** | **⭘** | **⭘** | **⭘** | **⭘** | **⭘** | **⭘** | **⭘** | **⭘** | **⭘** | **⭘** | **⭘** |
| **3.2 Results:**  **Figure 2: Projected development of the number of persons in need of long-term care and use of selected care services in the Rhine-Neckar district from 2014 to 2035** | **⭘** | **⭘** | **⭘** | **⭘** | **⭘** | **⭘** | **⭘** | **⭘** | **⭘** | **⭘** | **⭘** |
| **3.3 Results:**  **Figure 3: Projected development of the number of people with dementia in the Rhine-Neckar district from 2014 to 2035** | **⭘** | **⭘** | **⭘** | **⭘** | **⭘** | **⭘** | **⭘** | **⭘** | **⭘** | **⭘** | **⭘** |
| **3.4 Results: Textual description** | **⭘** | **⭘** | **⭘** | **⭘** | **⭘** | **⭘** | **⭘** | **⭘** | **⭘** | **⭘** | **⭘** |
| **4. Discussion and conclusions** | **⭘** | **⭘** | **⭘** | **⭘** | **⭘** | **⭘** | **⭘** | **⭘** | **⭘** | **⭘** | **⭘** |

1. **Out of 1,000 people in a small town 500 are members of a choir. Out of these 500 members in the choir 100 are men. Out of the 500 inhabitants that are not in the choir 300 are men. What is the probability that a randomly drawn man is a member of the choir?**

*Only numbers may be entered in this field.*

*Please enter your answer here:*

1. **Imagine we are throwing a five-sided die 50 times. On average, out of these 50 throws how many times would this five-sided die show an odd number (1, 3 or 5)?**

*Only numbers may be entered in this field.*

*Please enter your answer here:*

1. **Imagine we are throwing a loaded die (6 sides). The probability that the die shows a 6 is twice as high as the probability of each of the other numbers. On average, out of these 70 throws, how many times would the die show *the* number 6?**

*Only numbers may be entered in this field.*

*Please enter your answer here:*

1. **In a forest 20% of mushrooms are red, 50% brown and 30% white. A red mushroom is poisonous with a probability of 20%. A mushroom that is not red is poisonous with probability of 5%. What is the probability that a poisonous mushroom in the forest is red?**

*Only numbers may be entered in this field.*

*Please enter your answer here:*

1. Author’s note: Questions no. 14 to 17 relate to figure 1. In the computer-assisted version of this questionnaire, questions no. 14 to 17 were displayed together only with figure 1. [↑](#footnote-ref-1)
2. Author’s note: Question no. 18 relates to figure 2. In the computer-assisted version of this questionnaire, question no. 8 was displayed together only with figure 2. [↑](#footnote-ref-2)
