# Supplementary material for: Use of a quantitative data report in a hypothetical decision scenario for health policymaking: a computer-assisted laboratory study
Source: BMC Med Inform Decis Mak. 2021 Jan 28;21:32. doi: 10.1186/s12911-021-01401-4 (PMC7845041; doi:10.1186/s12911-021-01401-4)
Supplement: Supplementary file 3 — Additional file 3. Interview guide. [file 12911_2021_1401_MOESM3_ESM.docx]

Additional file 3: Interview Guide

(translated from German to English language)

Introduction

- Thank you for participation so far
- Presentation of the study part: How to deal with the report and the associated task (decision between three options)
- Procedure: Conversation circa 20 minutes, conversation about personal impressions and views, therefore there are neither right nor wrong answers
- Confidentiality and data security: tape recording (show device), all personal data will be made anonymous, transcription - then delete the tape recording, repeatedly point out the voluntary nature of participation, repeatedly obtain consent (verbally)
- Questions of the interview partner

>>TEST RECORDING

>>START RECORDING

**Introductory question: What is it actually like - independent of today - what points of contact have you had with such reports so far?**

| 1 | **When you read the report earlier - what was your first impression?** |
| --- | --- |

- What did you like about it?

*(What other example comes to your mind? / What do you mean by that exactly?)*

- What did you like less?

*(What other example comes to your mind? / What do you mean by that exactly?)*

| 2 | **One can approach such a report in many different ways. For example, some people only read the conclusion. How did you do that earlier?** |
| --- | --- |

- Which parts of the report did you pay special attention to?

*(Why did you proceed in this way? / What do you mean exactly?)*

- How extensively did you deal with texts?

*(Why did you proceed in this way? / What do you mean exactly?)*

- How extensively have you dealt with graphics?

*(Why did you proceed in this way? / What do you mean exactly?)*

- And how else do you proceed when reading a data report? Like today?

| 3 | **When you read the report earlier, you had to choose one of three options. How did you consider the report in your decision?** |
| --- | --- |

- Which information from the report was important for your decision?

*(Which text section/illustration exactly? / Why exactly was it important for you?)*

- Which information was less important?

*(Which section/illustration do you mean exactly? / Why was that less / not important for you?)*

- When did you decide on this option?

*(e.g. before you read the report, after you read a part / After which part of the report was that? / What do you think - why did you decide then?)*

- What - independent of the report - was included in your decision

making?

*(Can you explain this in more detail? / What else was important to you in your decision?)*

| 4 | **If you could give three suggestions to people who produce such data reports for the next reports - what would they be?** |
| --- | --- |

- What information do you like to have in reports?

*(What kind of key figures? / What about information on uncertainty? / What kind of statements? / Why?)*

- How should this information be presented?

*(How detailed? / What about text? / What about graphics? / What types of graphics? / What about tables? / Why?)*

**Final question: Now I have a very last question: Is there anything to report that we have not mentioned but you would like to tell us?**

>> FINISH RECORDING

- Thank you very much for taking the time for this interview!
