# Supplementary material for: Use of a quantitative data report in a hypothetical decision scenario for health policymaking: a computer-assisted laboratory study
Source: BMC Med Inform Decis Mak. 2021 Jan 28;21:32. doi: 10.1186/s12911-021-01401-4 (PMC7845041; doi:10.1186/s12911-021-01401-4)
Supplement: Supplementary file 4 — Additional file 4. Heatmaps of all 46 participants. [file 12911_2021_1401_MOESM4_ESM.docx]

**
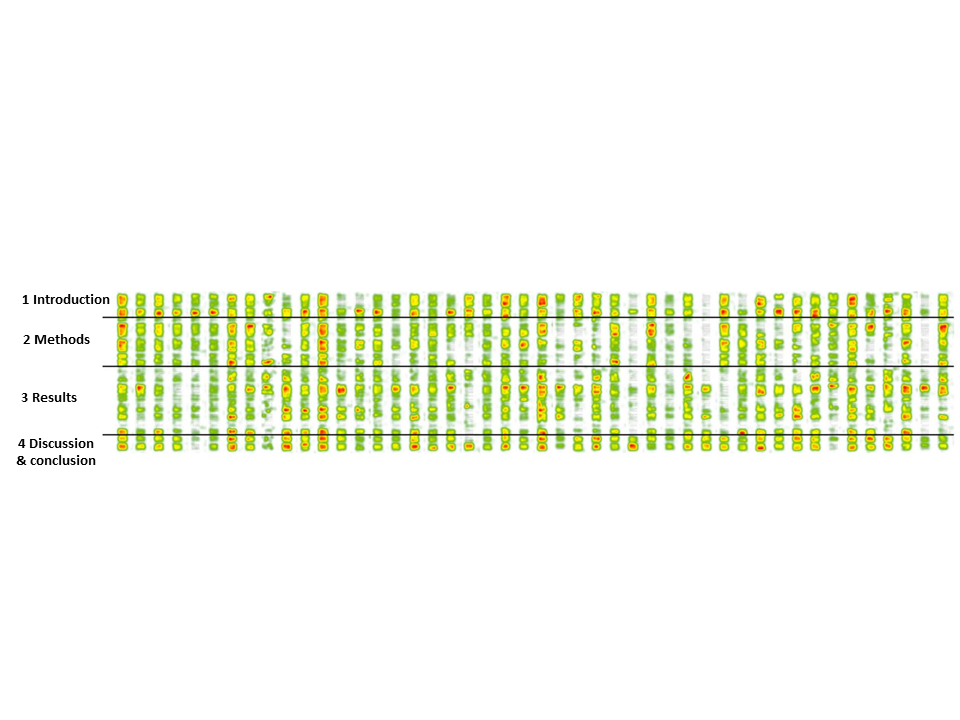
Heatmaps of all 46 participants (columns) after reading the data report (rows), scaled by fixation duration**

Average fixation duration in seconds over the 11 participants whose heatmaps are displayed above: red: 0.31 - more / yellow: 0.30 - 0.24 / green 0.23-less.
