# Supplementary material for: Use of a quantitative data report in a hypothetical decision scenario for health policymaking: a computer-assisted laboratory study
Source: BMC Med Inform Decis Mak. 2021 Jan 28;21:32. doi: 10.1186/s12911-021-01401-4 (PMC7845041; doi:10.1186/s12911-021-01401-4)
Supplement: Supplementary file 5 — Additional file 5. Graphics: Average fixation duration and time spent per report section. [file 12911_2021_1401_MOESM5_ESM.docx]

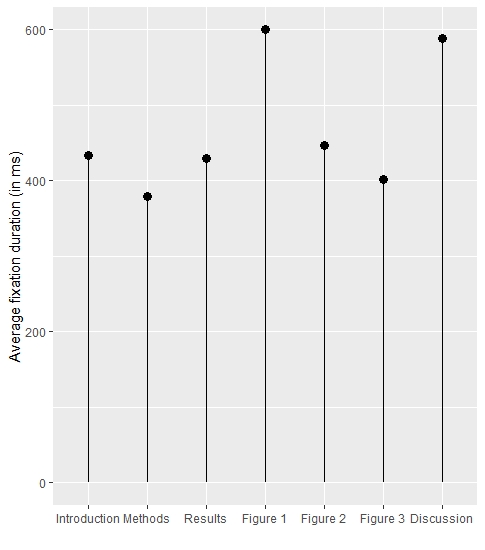


Figure 1: Average fixation duration (in milliseconds) per report section (n=46)


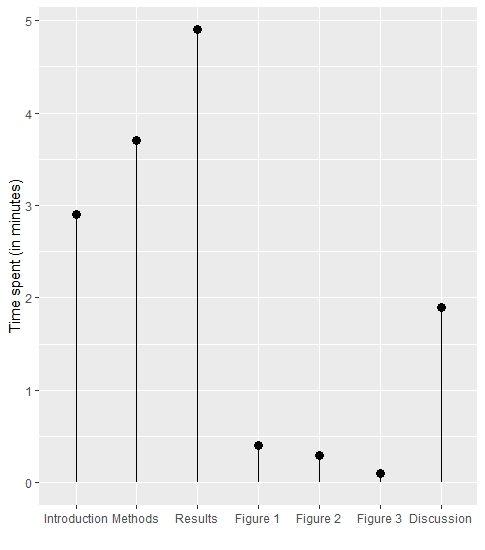


Figure 2: Time spent (in minutes) per report section (n=46)
